# Genome-wide characterization and expression analysis of α-amylase and β-amylase genes underlying drought tolerance in cassava

**DOI:** 10.1186/s12864-023-09282-9

**Published:** 2023-04-06

**Authors:** Taiyi Yang, Hengrui Li, Liangwu Li, Wanling Wei, Yuanhang Huang, Faqian Xiong, Maogui Wei

**Affiliations:** 1 College of Agronomy, Guangxi University, Nanning, 530004 China; 2 Guangxi South Subtropical Agricultural Sciences Research Institute, Chongzuo, 532406 China; 3 Sugarcane Research Institute, Guangxi Academy of Agricultural Sciences, Nanning, 530007 China; 4 State Key Laboratory for Conservation and Utilization of Subtropical Agro-bioresources, Guangxi University, Nanning, 530004 China; 5 Guangxi Key Laboratory of Agro-environment and Agro-products Safety, Guangxi University, Nanning, 530004 Guangxi China

**Keywords:** Cassava, Amylase, Abiotic stress, Bio-informatics, Gene expression

## Abstract

**Background:**

Starch hydrolysates are energy sources for plant growth and development, regulate osmotic pressure and transmit signals in response to both biological and abiotic stresses. The α-amylase (AMY) and the β-amylase (BAM) are important enzymes that catalyze the hydrolysis of plant starch. Cassava (*Manihot esculenta* Crantz) is treated as one of the most drought-tolerant crops. However, the mechanisms of how AMY and BAM respond to drought in cassava are still unknown.

**Results:**

Six *MeAMY* genes and ten *MeBAM* genes were identified and characterized in the cassava genome. Both *MeAMY* and *MeBAM* gene families contain four genes with alternative splicing. Tandem and fragment replications play important roles in the amplification of *MeAMY* and *MeBAM* genes. Both MeBAM5 and MeBAM10 have a BZR1/BES1 domain at the N-terminus, which may have transcription factor functions. The promoter regions of *MeAMY* and *MeBAM* genes contain a large number of cis-acting elements related to abiotic stress. *MeAMY1*, *MeAMY2*, *MeAMY5*, and *MeBAM3* are proven as critical genes in response to drought stress according to their expression patterns under drought. The starch content, soluble sugar content, and amylase activity were significantly altered in cassava under different levels of drought stress.

**Conclusions:**

These results provide fundamental knowledge for not only further exploring the starch metabolism functions of cassava under drought stress but also offering new perspectives for understanding the mechanism of how cassava survives and develops under drought.

**Supplementary Information:**

The online version contains supplementary material available at 10.1186/s12864-023-09282-9.

## Background

Starch hydrolysates are energy sources for plant growth and development, which also regulate osmotic pressure and transmit signals in response to biological and abiotic stresses [1, 2]. Amylase is the key enzyme during the starch hydrolysis process and both α-amylase (EC3.2.1.1, AMY) and β-amylase (EC3.2.1.2, BAM) are members of the glycoside hydrolase family. As an endo-amylase, AMY catalyzes the hydrolysis of the 1,4-glycosidic links, breaks the 1,4-glycosidic bonds inside the molecular and thus hydrolyzes amylose, amylopectin, or glycogen. BAM is an exo-amylase that progressively removes maltose or glucose from the non-reducing end by acting on the 1,4-glycosidic bond of amylose, amylopectin, and glycogen. Maltose, glucose, and dextrin are the main hydrolysates [1, 3]. AMY and BAM are classified into the glycoside hydrolase family 13 (GH 13) and GH 14 by Cazy (http://www.cazy.org/), respectively, and GH13 includes several isozymes. Their conserved domains can be queried within the Pfam database (http://pfam.xfam.org/). Based on the *AMY* and *BAM* gene family members and major structural features of the model plants (rice and Arabidopsis), the dominant conserved domains of the *AMY* and *BAM* gene families are found as Alpha-amylase (PF00128) and Glyco_hydro_14 (PF01373), respectively [4].

As the starch hydrolysates in plants, soluble sugar can alleviate the adverse effects of stress-induced carbon consumption when the synthesis of carbs is hindered [5] and also play significant roles in cell osmotic adjustment, acting like the essential osmotic protection agent and compatible solute [6]. The lack of sugar or energy of rice under anoxic circumstances during the seeding stage increased the expression of the *Amy3* subfamily gene [7]. In Arabidopsis leaves, the *AtAMY1* (At4g25000) gene was up-regulated under biotic and abiotic stresses and the corresponding AMY showed high heat tolerance [8]. The *AtBAM1* mutant *bam1* showed higher tolerance to osmotic and drought stress than the control [9, 10]. The expressions of *AtBAM1* and *AtBAM3* genes increased the maltose content in Arabidopsis leaves, which relieved the heat stress to a certain extent by protecting their photosynthetic electron transport chains [11, 12]. Poor root systems of Arabidopsis double mutants under osmotic stress were found, which *AMY3* and *BAM1* genes were missing [13]. Under drought stress, soybean leaves contained less starch and more soluble sugars than control, while the expression levels of *GmAMY3* and *GmBAM1* also altered dramatically [14]. Furthermore, overexpression of *VvBAM1* improved tomato cold tolerance by modulating starch hydrolysis and scavenging reactive oxygen species (ROS) in plants [15].

Cassava is widely grown in tropical and subtropical regions because of its starchy root and is treated as one of the most drought-tolerant crops [16, 17]. Although *AMY* and *BAM* genes have been identified in other plants, only two genes of the *MeAMY* and *MeBAM* families were cloned and characterized from cassava leaves [18]. The biological roles of the *MeAMY* and *MeBAM* families in cassava under stress are still unknown. Thus, *MeAMY* and *MeBAM* genes were identified in the current study. Characterization, multiple sequence alignment, phylogenetic analysis, conserved structural motif, gene structure analysis, chromosomal distribution, homologs, collinearity analysis, and cis-regulatory element prediction of these two genes were performed. Additionally, the expression patterns of the *MeAMY* and *MeBAM* families under drought stress were also analyzed.

## Results

### Characterization and prediction of the subcellular localization of *MeAMY* and *MeBAM* genes

To identify protein-coding genes which contain the specific domain, the whole genome was scanned using BLASTP and HMMER. In total, six *MeAMY* genes and ten *MeBAM* genes were identified and renamed. Their characteristics and subcellular localization information were all listed in Table 1. Homology analysis was conducted on 13 transcripts of *MeAMY* and 20 transcripts of *MeBAM*, respectively (Fig. S1). The size of *MeAMY* genes ranged from 1227 ~ 2691 bp and the similarities among sequences ranged from 0.53 to 1.00 (Fig. S1a). The length of MeAMY protein sequence ranged from 408 to 896 aa and their similarities ranged from 0.44 ~ 1.00, while their theoretical pIs ranged from 4.71 to 8.22 (Table 1). The range of molecular mass and aliphatic index were 46.560 ~ 101.705 kDa and 27.07 ~ 45.39, respectively. Proteins were all hydrophilic with a negative GRAVY (− 0.576 ~ − 0.226) and their instability index ranged from 27.07 to 45.39. MeAMY1.1 ~ MeAMY3.5 were unstable with an instability index larger than 40, while the other four sequences were shown as stable proteins.

The size of *MeBAM* genes ranged from 1227 ~ 2691 bp and their similarities were 0.41 ~ 1.00 (Fig. S1b). The length of MeBAM protein sequence ranged from 429 to 701 aa and their similarities were 0.27 ~ 1.00, while their theoretical pIs ranged from 5.3 to 8.92. The range of molecular mass and aliphatic index were 48.937 ~ 79.132 kDa and 69.6 ~ 84.41, respectively. Proteins were all hydrophilic with a negative GRAVY (− 0.485 ~ − 0.249) and their instability index range was 31.44 ~ 52.03. MeBAM1, MeBAM2, MeBAM5.1 ~ MeBAM5.6, MeBAM8, MeBAM10.1, and MeBAM10.2 were unstable, while the other nine sequences were shown as stable proteins.

The prediction of the subcellular localization of 13 *MeAMY* and 20 *MeBAM* genes was conducted using CELLO v.2.5 and the results were listed in Table 1. *MeAMY1* and *MeAMY2* are located in the cytoplasm, while other *MeAMY* genes located in the Extracellular. *MeBAM1* and *MeBAM2* were located in chloroplast, while *MeBAM3* and *MeBAM9* were found in mitochondria, *MeBAM4* ~ *MeBAM7* in the cytoplasm, *MeBAM8* in the plasma membrane, and *MeBAM10* in the nucleus.

**Table 1:** Characteristics and prediction of subcellular localization of the MeAMY and MeBAM proteins

Gene name	protein Accession No.	Length of AA	PI	MW(Da)	GRAVY	Aliphatic index	Instability index	Subcellular localization
*MeAMY1.1*	XP_021603448.1	896	6.3	101705.99	-0.502	74.08	41.72	Cytoplasmic
*MeAMY1.2*	XP_021603456.1	812	6.32	91803.7	-0.529	74.45	44.78	Cytoplasmic
*MeAMY2.1*	XP_021611463.1	896	5.67	101574.29	-0.523	75.29	41.35	Cytoplasmic
*MeAMY2.2*	XP_021611464.1	812	5.63	91815.24	-0.538	76.49	45.39	Cytoplasmic
*MeAMY3.1*	XP_021612774.1	408	8.22	46560.08	-0.576	68.85	42.85	Extracellular
*MeAMY3.2*	XP_021612775.1	408	8.22	46560.08	-0.576	68.85	42.85	Extracellular
*MeAMY3.3*	XP_021612776.1	408	8.22	46560.08	-0.576	68.85	42.85	Extracellular
*MeAMY3.4*	XP_021612777.1	408	8.22	46560.08	-0.576	68.85	42.85	Extracellular
*MeAMY3.5*	XP_021612778.1	408	8.22	46560.08	-0.576	68.85	42.85	Extracellular
*MeAMY4*	XP_021625997.1	429	5.1	48230.1	-0.311	84.13	31.49	Extracellular
*MeAMY5*	XP_021631425.1	424	4.71	46962.8	-0.226	83.99	29.81	Extracellular
*MeAMY6.1*	XP_021601526.1	762	4.89	84901.46	-0.358	76.19	31.03	Extracellular
*MeAMY6.2*	XP_021601463.1	430	4.92	48130.31	-0.279	79.63	27.07	Extracellular
*MeBAM1*	XP_021605553.1	569	8.59	64184.29	-0.303	73.88	42.52	Chloroplast
*MeBAM2*	XP_021608087.1	581	5.67	64970.59	-0.388	71.2	41.1	Chloroplast
*MeBAM3*	XP_021608216.1	535	6.04	59219.83	-0.344	76.02	37.5	Mitochondrial
*MeBAM4.1*	XP_021611624.1	545	5.69	61669.84	-0.281	75.69	37.54	Cytoplasmic
*MeBAM4.2*	XP_021611626.1	517	5.59	58563.38	-0.315	77.72	35.62	Cytoplasmic
*MeBAM4.3*	XP_021611627.1	429	5.52	48937.51	-0.346	75.94	32.57	Cytoplasmic
*MeBAM5.1*	XP_021613139.1	701	5.63	79132.18	-0.47	76.5	40.8	Cytoplasmic
*MeBAM5.2*	XP_021613140.1	701	5.63	79132.18	-0.47	76.5	40.8	Cytoplasmic
*MeBAM5.3*	XP_021613141.1	701	5.63	79132.18	-0.47	76.5	40.8	Cytoplasmic
*MeBAM5.4*	XP_021613142.1	701	5.63	79132.18	-0.47	76.5	40.8	Cytoplasmic
*MeBAM5.5*	XP_021613143.1	701	5.63	79132.18	-0.47	76.5	40.8	Cytoplasmic
*MeBAM5.6*	XP_021613144.1	697	5.63	78691.58	-0.485	75.97	40.7	Cytoplasmic
*MeBAM6.1*	XP_021630045.1	594	6.04	67689.21	-0.432	84.41	37.18	Cytoplasmic
*MeBAM6.2*	XP_021630046.1	521	5.3	59078.17	-0.413	82.59	31.44	Cytoplasmic
*MeBAM7*	XP_021594996.1	582	6.05	64837.42	-0.42	69.6	36.57	Cytoplasmic
*MeBAM8*	XP_021595073.1	522	8.92	59418.2	-0.249	82.2	52.03	Plasma Membrane
*MeBAM9*	XP_021594434.1	546	8.7	61339.7	-0.459	70.04	34.79	Mitochondrial
*MeBAM10.1*	XP_021594905.1	691	5.51	77653.08	-0.446	71.74	40.13	Nuclear
*MeBAM10.2*	XP_021594906.1	689	5.44	77325.77	-0.402	73.5	40.35	Nuclear
*MeBAM10.3*	XP_021594907.1	686	5.5	77103.67	-0.394	73.67	39.9	Nuclear

### Structure and alternative splicing of *MeAMY* and *MeBAM* genes with analyses of conserved domains and motifs of their proteins

Comparing the domains of the AtAMY1 and AtBAM1 proteins, similar conserved areas of the MeAMY and MeBAM protein sequences were identified. Two carbohydrate binding sites and three catalytic residues were found in MeAMY family (Fig. S2a) and these amino acid sites were perfectly conserved. Additionally, the MeBAM family has two catalytic residues Glu-186 and Glu-380. The amino acid at Glu-186 was highly conserved, while Glu-380 showed poor conservation of amino acids. A mutation at Glu-380 occurred in five different sequences and it was changed to glutamine (Gln), arginine (Arg), and valine (Val) in MeBAM3, in MeBAM8, and in MeBAM10.1 ~ 10.3, respectively (Fig. S2b). The MeBAM contained 20 carbohydrate binding sites and two of them had highly conserved amino acids, while 18 carbohydrate binding sites had amino acid mutations. Both the amino acids in the flexible loop and in the inner loop showed poor conservation with a large number of mutations.

**Fig. 1:**
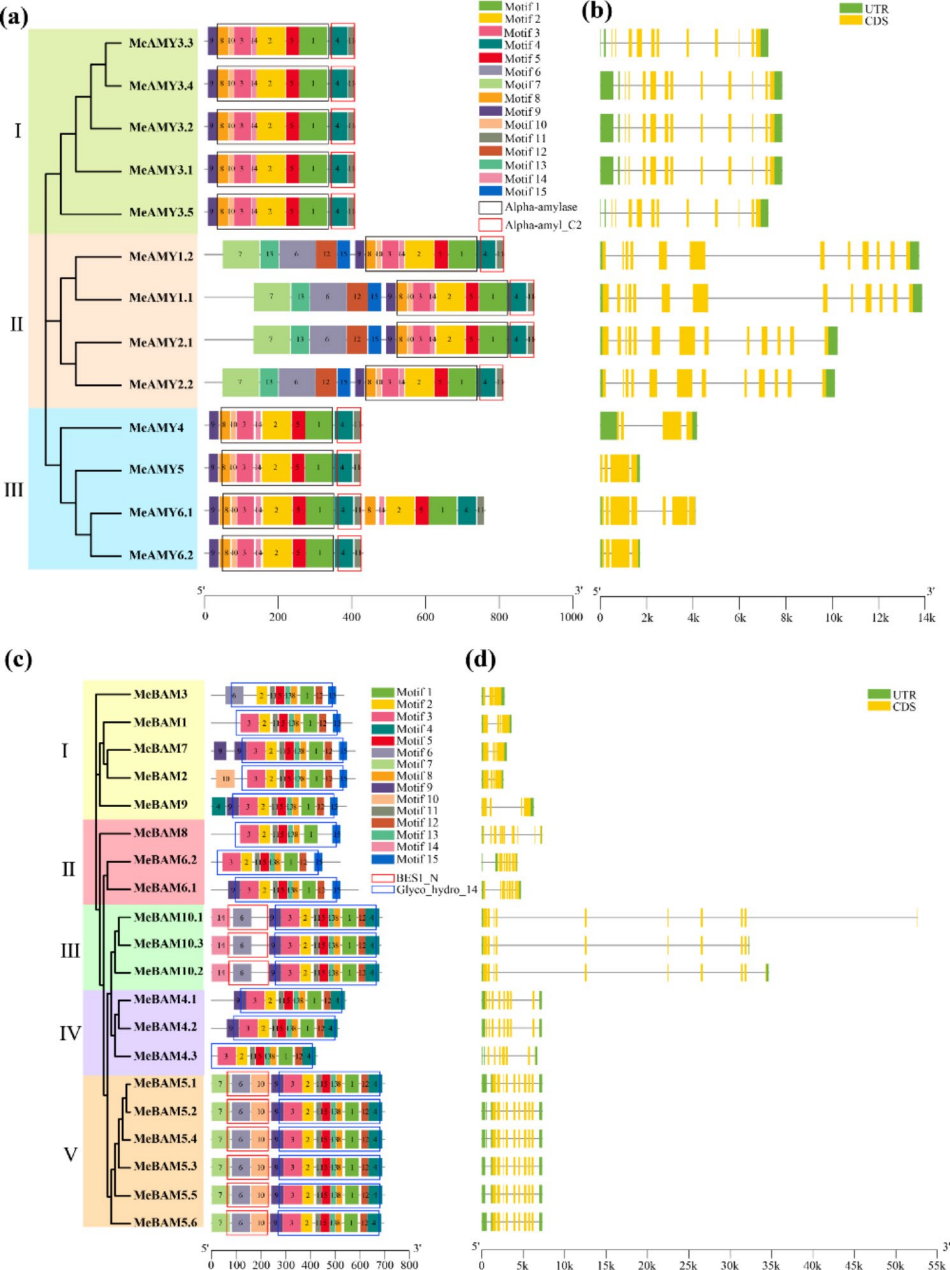
Bioinformatics analyzes of *MeAMY* and *MeBAM* gene families. **a**: Evolutionary tree, conserved motifs, and domains of MeAMY families. **b**: Gene structure of *MeAMY* genes family. **c**: Evolutionary tree, conserved motifs, and domain of MeBAM families. **d**: Gene structure of *MeBAM* genes family. The black, red, and blue boxes in **a** and **c** represent the conserved domains

To better understand the structural evolution of *MeAMY* and *MeBAM*, the unrooted evolutionary trees were constructed based on their protein sequences. For MeAMY, 13 MeAMY were classified into three clusters I ~ III and a total of 15 conserved motifs of MeAMY were identified using MEME software (Fig. 1a). The cluster I contains five *MeAMY* genes which are alternative splicing of one gene and they shared the same conserved motifs (Fig. 1b). The four genes in Cluster II shared the same conserved motifs. *MeAMY1.1* and *MeAMY2.1* belonged to alternate donor site (ADS) and all had a CDS sequence of 252 bp longer than *MeAMY1.2* and *MeAMY2.2*, respectively. Motif 6, 7, 12, 13, and 15 were only observed in Cluster II. Cluster III contains MeAMY4, MeAMY5, MeAMY6.1, and MeAM6.2. MeAMY6.1 has the largest number of conservative motifs and motif 3, 9, and 10 were missing in the repeat, which indicated that MeAMY6.1 is repeat motifs. In addition, the 5’ and 3’ ends of *MeAMY4* have the longest UTR in cluster III (Fig. 1a and b).

The MeBAM was divided into five clusters (Fig. 1c). The distribution of conserved motifs at the 5’ end of *MeBAM* in cluster I was inconsistent. The positions and arrangements of their CDS and UTR were also different (Fig. 1d). In cluster II, *MeBAM6.1* and *MeBAM6.2* were alternative splicing of the same gene and treated as alternate promoters (AP). The *MeBAM10.1* ~ *MeBAM10.3* genes in cluster III shared same conserved motifs and belonged to alternate terminators (AT). Their UTR and CDS sequences of the 3’ end were different. In cluster IV, *MeBAM4.1*, *MeBAM4.2*, and *MeBAM4.3* can be treated as AP. The six genes in cluster V shared same conserved motifs and CDS.

### Chromosomal location and collinearity analysis of *MeAMY* and *MeBAM*

The *MeAMY* and *MeBAM* genes were found in eight chromosomes according to the Generic Feature Forma file of the cassava genome (Fig. S3). For *MeAMY*, only *MeAMY2* was located in the ‘+’ strand, while others were located in the ‘−’ strand. *MeBAM1*, *MeBAM4*, *MeBAM5*, *MeBAM7*, and *MeBAM10* were located in the ‘+’ strand, while the remaining *MeBAM* genes were located in the ‘−’ strand.

**Fig. 2:**
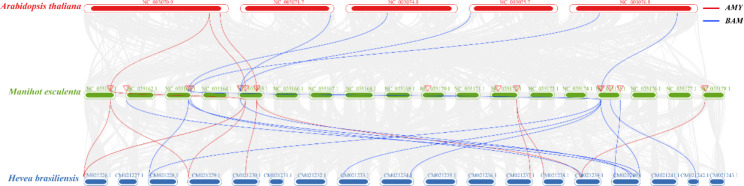
The collinear relationships of *AMY* and *BAM* genes in *Manihot esculenta*, *Arabidopsis thaliana* and *Hevea brasiliensis*

In order to reveal the expansion and evolution mechanisms of *MeAMY* and *MeBAM* gene families, the collinearities of coding genes and the potential gene duplication events in cassava genome were analyzed. Combined with the Arabidopsis genome and the rubber tree (*Hevea brasiliensis*) genome, the collinearities of coding genes among different species were also analyzed (Fig. 2, Table S1). There were linear relationships between *MeAMY1* and *MeAMY2*, *MeBAM4* and *MeBAM5*, and *MeBAM2* and *MeBAM7*, respectively. *MeBAM4* and *MeBAM5* were tandem repeat genes. The Ka/Ks values of the flanking homologous genes of those three pair genes with linear relationships of the *MeAMY* and *MeBAM* families ranged from 0.181848632 to 0.30120533, suggesting that their divergence was driven by purifying selection (Table 2; Fig. 2).

Furthermore, there were eight pairs of genes with collinearity between the cassava genome and the Arabidopsis genome, including three pairs of *AMY* genes and five pairs of *BAM* genes. There were 23 pairs of genes, 10 *AMY* genes and 13 *BAM* genes, in collinearity between the cassava genome and the rubber tree genome.

**Table 2:** Homologous gene Ka/Ks values of *MeAMY* and *MeBAM* gene families

Seq-1	Seq-2	Ka	Ks	Ka/Ks
*MeAMY1.1*	*MeAMY2.1*	0.080411456	0.266965581	0.30120533
*MeBAM2*	*MeBAM7*	0.072314402	0.39766261	0.181848632
*MeBAM4.3*	*MeBAM5.6*	0.231696907	1.021698702	0.226776159

### Phylogenetic analysis of MeAMY and MeBAM proteins

**Fig. 3:**
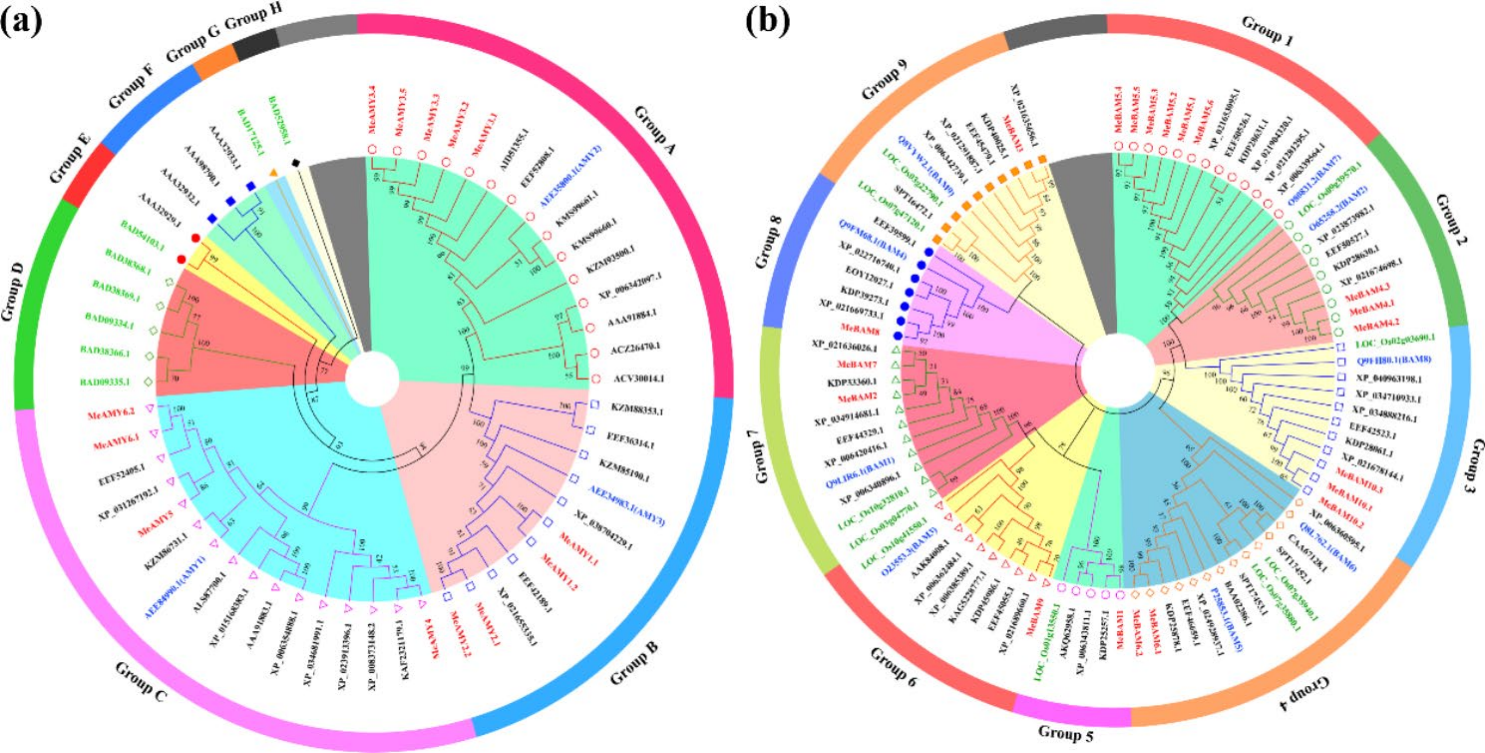
The phylogenetic tree of AMY (**a**) and BAM (**b**) proteins in *Manihot esculenta* and other species

The AMY and BAM protein sequences of other species were downloaded from NCBI database and nine rice BAM protein sequences were downloaded from Rice Genome Annotation Project (Table S2). A neighbor-joining tree was constructed based on the alignments of proteins of AMY and BAM families from cassava, rice, Arabidopsis, and other species. In total, 54 AMY proteins from 12 species were used to construct the phylogenetic tree and classified into eight groups, including monocotyledonous plants (Group A ~ C) and dicotyledonous plants (Group D ~ H) (Fig. 3a). The cassava AMYs were classified into Group A, B, and C and closely related with rubber trees and castors (*Ricinus communis*), which were all Euphorbiaceae plants.

The BAM protein sequences were divided into nine groups. Each group contains both the BAM protein sequences of monocots and dicots (Fig. 3b). The BAM protein sequences of Arabidopsis, rice, and cassava were evenly distributed in all groups, while the BAM protein sequences of rubber trees, castors, and Jatropha (*Jatropha curcas*) were close to MeBAM among all groups.

### Cis-acting elements in the promoter regions of *MeAMY* and *MeBAM* genes

**Fig. 4:**
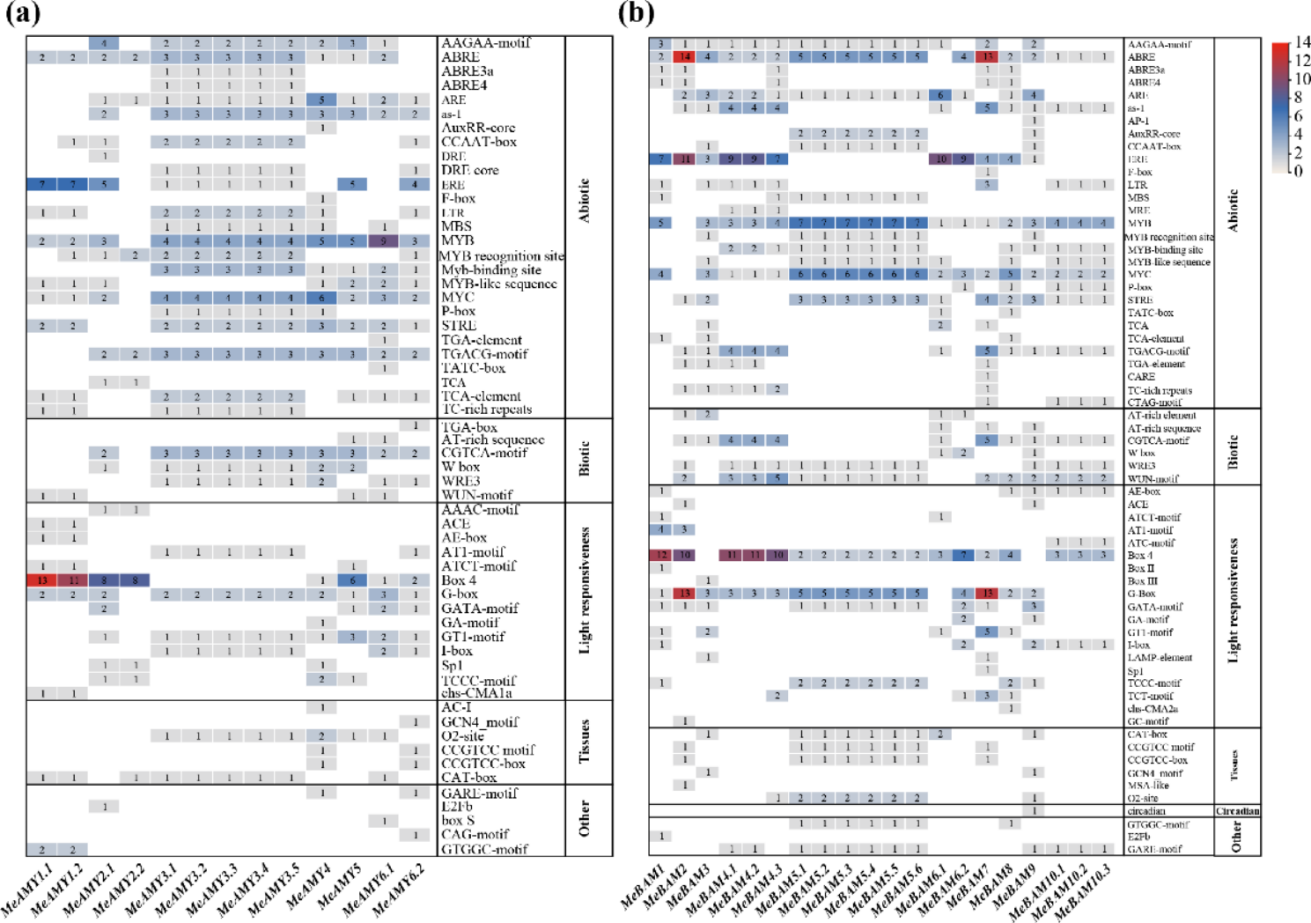
Distribution of cis-acting elements of *MeAMY* (**a**) and *MeBAM* (**b**) genes. Number in color blocks represents the number of cis-acting elements

In order to understand how the *MeAMY* and *MeBAM* gene families respond to stress, the cis-acting elements in their promoter regions were also analyzed (Fig. S4). The core promoter elements CAAT-box, TATA-box, A-box, and AT ~ TATA-box were first removed in the promoter regions of *MeAMY* and *MeBAM* genes and cis-acting elements were then classified according to Yue method [1]. The number of cis-acting elements responding to abiotic stress is the largest among all the cis-acting elements, such as MYB, TGACG-motif, ERE, MYC, as-1, and ARE. In the promoter region of *MeAMY* gene, the number of MYB elements was the largest and 76.9% of the sequences contained 2 ~ 3 CGTCA-motifs (Fig. 4a). There were nine MYB elements enriched in the promoter region of *MeAMY6.1*. There were seven ERE elements enriched in the promoter regions of both *MeAMY1.1* and *MeAMY1.2*, while six MYC elements were enriched in *MeAMY4* (Fig. 4a). For *MeBAM*, 80% of all the sequences contained WUN-motifs and there were five WUN-motifs enriched in the promoter region of *MeBAM4.3* (Fig. 4b). There were 14 ABRE elements and 13 ABRE elements enriched in *MeBAM2* and in *MeBAM7*, respectively. There seven MYB and six MYC elements enriched in *MeBAM5.1* ~ *MeBAM5.6*. ERE elements were enriched in the promoter regions of *MeBAM1*, *MeBAM2*, *MeBAM4.1, MeBAM4.2, MeBAM4.3*, *MeBAM6.1*, and *MeBAM6.2* genes.

Furthermore, the promoter regions of *MeAMY* and *MeBAM* genes also contained a large number of cis-acting elements related to light response, such as Box 4 and G-box (Fig. S4). Box 4 was the most abundant element, containing 50 elements in the promoter region of *MeAMY* gene. And 61.52% of the sequences contained this element, which enriched in *MeAMY1.1*, *MeAMY1.2*, *MeAMY2.1*, *MeAMY2.2*, and *MeAMY5*. And 90% of *MeBAM* genes contained 91 Box 4 elements, which were mainly enriched in *MeBAM1*, *MeBAM2*, *MeBAM4.1*, *MeBAM4.2*, and *MeBAM4.3*. In addition, there were 92.3% of *MeAMY* promoter region sequences included G-box elements, while 80% of *MeBAM* sequences contained G-box elements, which are mainly enriched in *MeBAM2* and *MeBAM7*.

### Gene ontology classification of MeAMY and MeBAM proteins

**Fig. 5:**
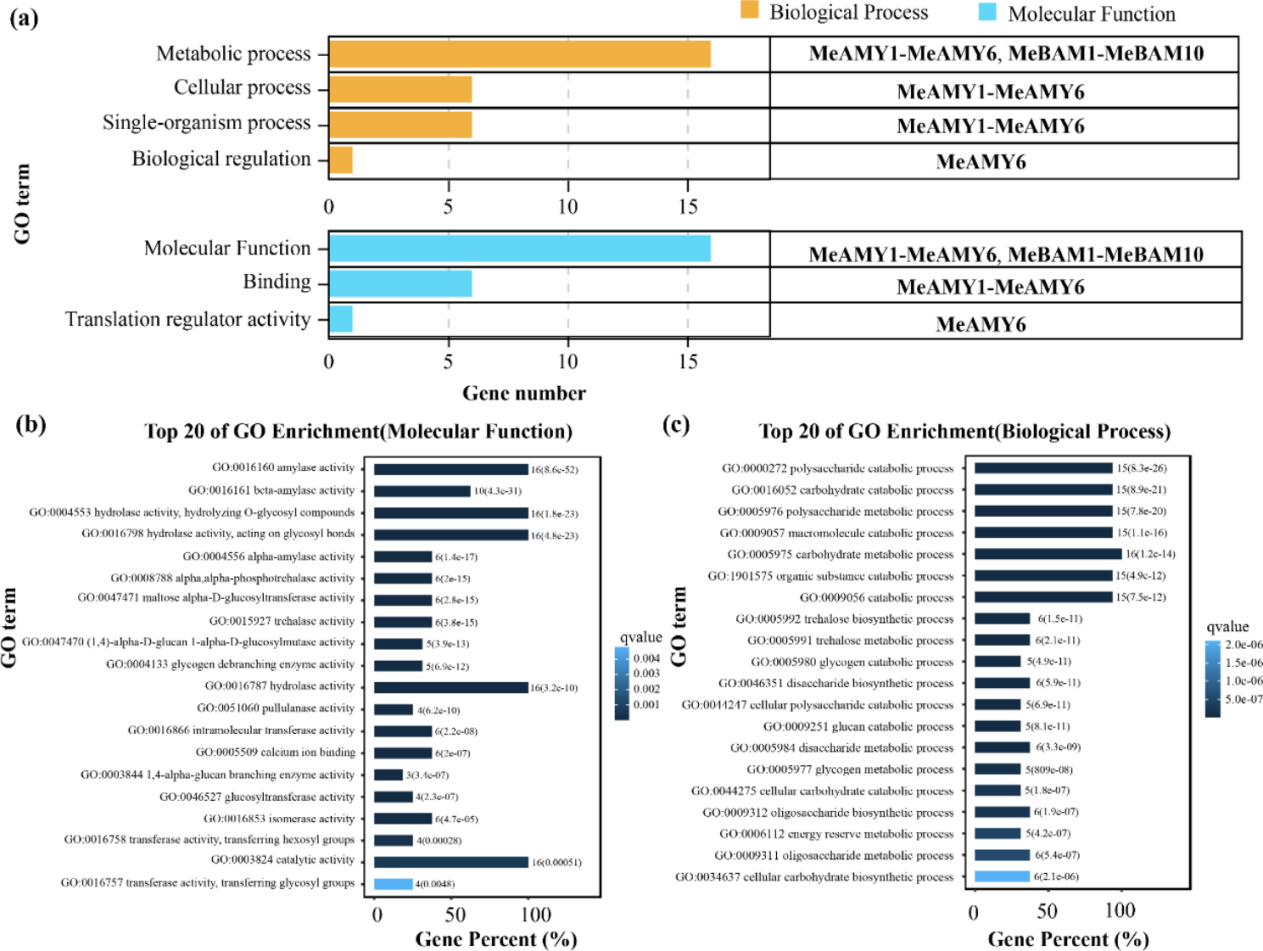
Gene ontology annotations of MeAMY and MeBAM proteins. **a**: overview of the GO annotations. **b**: molecular function of the top 20 GO terms. **c**: biological process of the top 20 GO terms

GO annotation of MeAMY and MeBAM proteins were separated into two categories: biological processes and molecular functions (Fig. 5a). MeAMY proteins were involved in both single-organism and cellular processes, while only MeAMY6 was involved in biological regulation. MeAMY and MeBAM proteins were further classified and then annotated to 111 GO IDs (Table S3). The molecular functions of MeAMY and MeBAM primarily included amylase activity, hydrolase activity, and catalytic activity (Fig. 5b). All MeAMY and MeBAM proteins were involved in carbohydrate metabolic process, polysaccharide catabolic process, polysaccharide metabolic process, macromolecule catabolic process, and organic substance catabolic process (Fig. 5c).

### Expression analysis of *MeAMY* and *MeBAM*

**Fig. 6:**
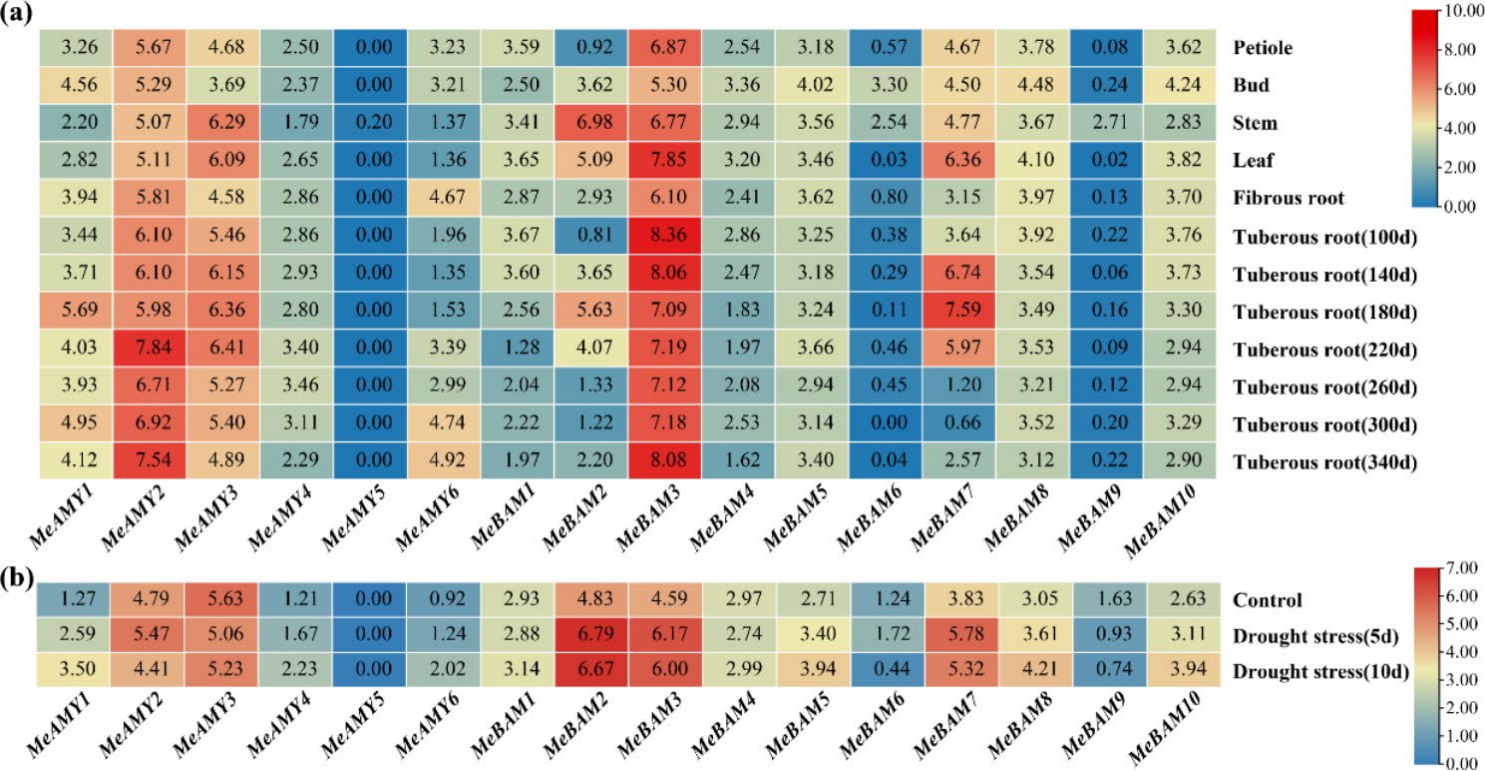
Expression profiles of *MeAMY* and *MeBAM* genes of cassava. **a**: samples collected under well watering conditions. **b**: cassava leaves under drought stress. Color scale represents transcripts per million reads (TPM) normalized log2(TPM + 1), where blue indicates low expression and red indicates high expression

The expression patterns of *MeAMY* and *MeBAM* were analyzed using RNA-seq data from various cassava tissues under well-watering conditions (Fig. 6a). *MeAMY2*, *MeAMY3*, and *MeBAM3* showed high expression levels in all studied tissues. *MeBAM2*, *MeBAM6*, and *MeBAM9* had the highest expression levels in 100-day-old stems among all collected samples, while both *MeBAM2* and *MeBAM7* displayed higher expression levels in leaves and *MeAMY6* was highly expressed in old roots. Under drought stress, the expression of *MeAMY1*, *MeAMY4*, *MeAMY6*, *MeBAM2*, *MeBAM3*, *MeBAM5*, *MeBAM7*, *MeBAM8*, and *MeBAM10* genes in cassava leaves were up-regulated, while the expression of *MeAMY3* and *MeBAM9* genes were down-regulated. The expression levels of *MeAMY2*, *MeBAM2*, *MeBAM3*, *MeBAM6*, and *MeBAM7* under the five days drought treatment were higher than the control and the ten days drought treatment (Fig. 6b).

qRT-PCR was used to analyze the expression of *MeAMY* and *MeBAM* genes in cassava during the seedling stage without drought stress (CK) and with drought stress. All of the genes were detected in all samples from CK (Fig. S5). *MeAMY5* and *MeBAM6* showed low expression levels in all tissues, while *MeBAM3* showed higher expressions. *MeBAM2* showed high expression in both leaves and stems, while *MeBAM7* showed high expression only in stems and *MeBAM9* was mainly expressed in leaves.

**Fig. 7:**
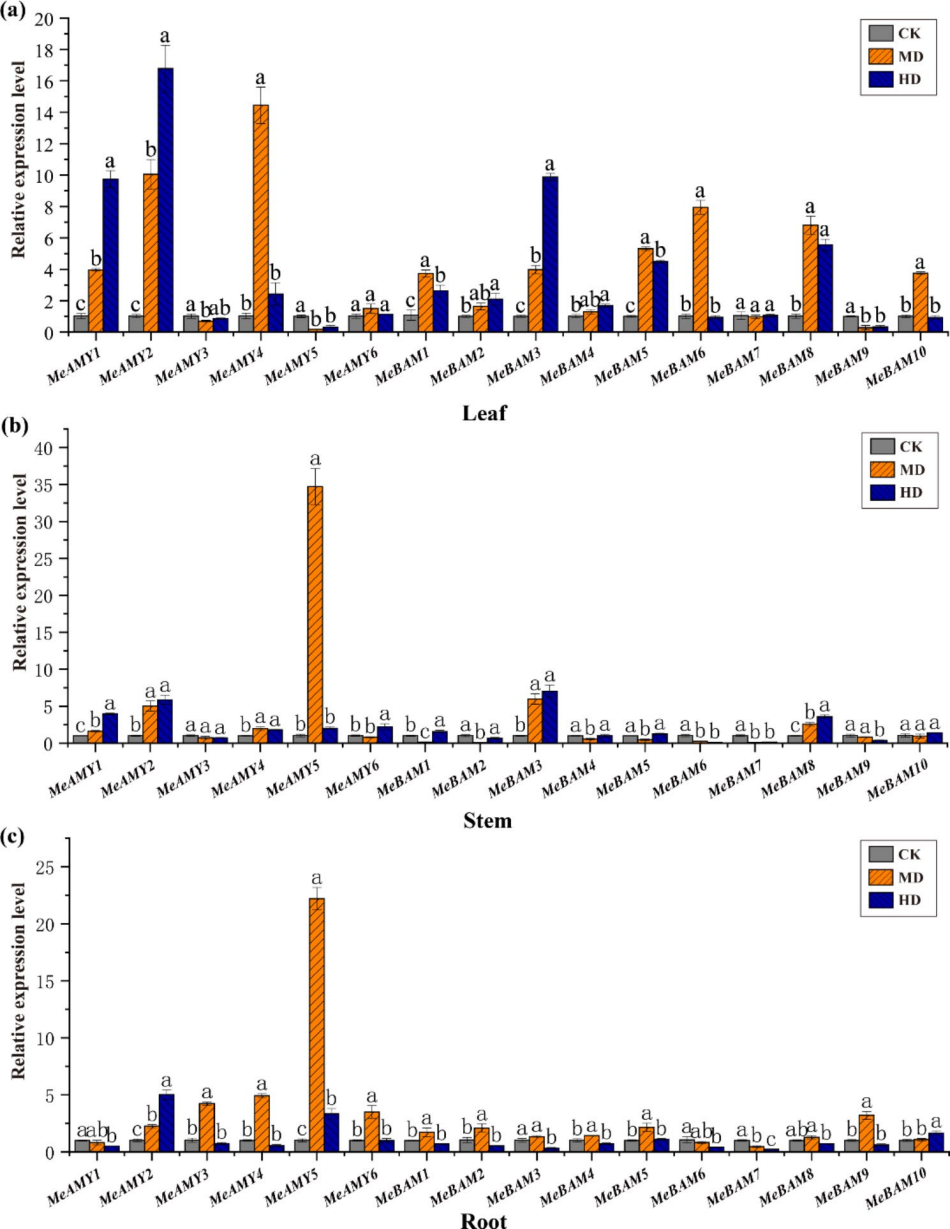
Expression patterns of *MeAMY* and *MeBAM* genes of cassava under drought stress. **a**: expression patterns in leaves; **b**: expression patterns in stems; **c**: expression patterns in roots. Expression levels were determined using qRT-PCR and calculated using the 2^−ΔΔCt^ method under the control of the *actin* housekeeping gene. Different letters indicate significant differences among different treatments of cassava (*P* < 0.05). CK, well watering; MD, the moderate drought; HD, the severe drought treatment

Compared to CK, the expression levels of *MeAMY1*, *MeAMY2*, *MeAMY4*, *MeBAM1*, *MeBAM3*, *MeBAM5*, *MeBAM6*, *MeBAM8*, and *MeBAM10* genes in leaves were up-regulated in the moderate drought (MD) treatment, whereas *MeAMY3*, *MeAMY5*, and *MeBAM9* genes showed down-regulated (Fig. 7a). *MeAMY5* gene was highly expressed in stem and root under moderate drought (Fig. 7b and c). *MeAMY1*, *MeAMY2*, and *MeBAM3* genes showed higher expression levels in leaves of the severe drought treatment (HD) than in the MD treatment, while the expression levels of *MeAMY4*, *MeBAM1*, *MeBAM5*, *MeBAM6*, *MeBAM8*, and *MeBAM10* genes decreased under HD treatment. Expression levels of *MeAMY1*, *MeAMY2*, *MeAMY4*, *MeAMY5*, *MeAMY6*, *MeBAM3*, and *MeBAM8* in stems increased with drought stress (Fig. 7b). *MeAMY3*, *MeAMY4*, *MeAMY5*, *MeAMY6*, *MeBAM1*, *MeBAM2*, *MeBAM3*, *MeBAM4*, *MeBAM5*, *MeBAM8*, and *MeBAM9* genes were highly up-regulated in root under the MD treatment, but down-regulated under the HD treatment (Fig. 7c).

### Variation of carbohydrate contents and amylase activity in cassava

**Fig. 8:**
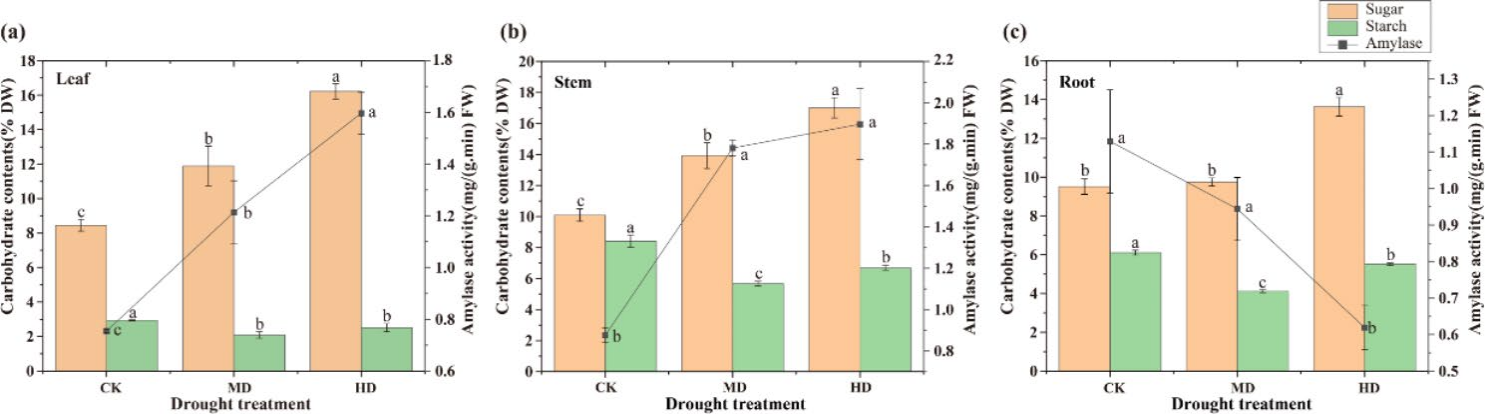
Soluble sugar and starch contents and the amylase activities of cassava under different water treatments. Amylase represents the total enzymatic activity of α-amylase and β-amylase. DW, dried weight; FW, fresh weight. CK, well watering; MD, the moderate drought; HD, the severe drought treatment

The soluble sugar content and the amylase activity in cassava leaves under HD treatment were higher than MD and CK, while its starch content was lower than CK and close to MD (Fig. 8a). Carbohydrates and amylase activity in stems varied similarly with leaves (Fig. 8b). In roots, the soluble sugar content of HD treatment was higher than MD and CK, while its starch content was lower than CK and slightly higher than MD (Fig. 8c). In contrast, the amylase activity of HD treatment was lower than MD and CK.

## Discussion

Previous studies on the *AMY* and *BAM* genes in quinoa, apple, barley, tea plants, and other plants revealed that they belonged to the multigene families [1, 19–21]. In the current study, six *MeAMY* genes and ten *MeBAM* genes were identified in the cassava genome. The composition of conserved motifs in MeAMY and MeBAM protein sequences were different. Some motifs only existed in a small number of gene family members causing differences among them and these differences related to the functional differentiation of *MeAMY* and *MeBAM* families during evolution. Additionally, alternative splicing was another crucial reason for the polymorphism of protein structure and function and transcripts of *MeAMY* and *MeBAM*. Both *MeAMY* and *MeBAM* gene families contain four genes with alternative splicing, *MeAMY1* and *MeAMY2* were on the same branch of the evolutionary tree (Fig. 1a) and their alternative splicing methods were also the same, ending up with similar transcripts. Their CDS are arranged similarly, located in the cytoplasm, and exhibit collinearity (Table 2), which indicates that they might have similar functions. In addition, *MeAMY3* in the *MeAMY* gene family produced five transcripts (Fig. 1a) and differences of these five transcripts are mainly in the 5’ UTR, leading to different expression patterns of these genes [22]. *MeAMY6* included two transcripts, which shared a high sequence similarity (0.99). MeAMY6.2 shares the same protein sequence as the former part (1 ~ 430 aa) of MeAMY6.1 and they both have a complete Alpha-amylase domain. GO annotation also proved that the MeAMY6 had translation regulation activity (Fig. 5a). In the *MeBAM* gene family, the *MeBAM10* gene formed into three transcripts and the last exon varied, which has the most obvious effect on the protein instability index (Table 1). The 3’ UTR of those three transcripts also varied, which control mRNA expression and translation, regulate mRNA stability through the rich AU element, and regulate protein-protein interactions [23].

The collinearity analysis results indicated that purification selection occurred of *MeAMY* and *MeBAM* and their protein sequence stabilities were somewhat preserved (Table 2) [24]. *MeBAM4* and *MeBAM5* were tandem replications (Fig. S3) according to the definition of tandem replication events by Recep Vatansever [25]. The amino acids of the five carbohydrate binding sites in these two gene sequences varied, which may lead to changes in their abilities to bind the substrate, ending up with different catalytic efficiencies of the substrate (Fig. S2). *MeAMY1* and *MeAMY2*, *MeBAM2* and *MeBAM7* were fragment replication (Fig. 2). There was no amino acid mutation and their carbohydrate binding sites and catalytic residues were exceedingly conservative.

The phylogenetic tree of the AMY proteins was divided into eight groups, including three dicotyledonous-plant groups and five monocotyledonous-plant groups (Fig. 3a), which is consistent with previous studies [1]. All the cassava AMYs were also classified into Group A, B, and C and the same as rubber trees and castors (*Ricinus communis*), demonstrating closer evolutionary relationships of the AMYs among Euphorbiaceae plants than other families (Fig. 3a). The AMY proteins of cassava and Arabidopsis in Groups A and B had collinearity (Fig. 2), sharing the same carbohydrate binding sites and catalytic residues (Fig. S2a). MeAMY3 and AtAMY2 were clustered into Group A, while MeAMY1, MeAMY2, and AtAMY3 were in Group B. MeAMY4, MeAMY5, and MeAMY6 were all found on the same branch as AtAMY1. Both AtAMY1 (AEE84990.1) and AtAMY2 (AEE35800.1) were secretory proteins and AtAMY3(AEE34983.1) was a special redox-regulated chloroplast α-amylase. Starch degradation could still be observed in the single, double, and triple knock-out mutant Arabidopsis leaves of these three genes, which indicated that α-amylase was not necessary during the transient starch decomposition process in Arabidopsis leaves [26]. Furthermore, AtAMY3 on Group B could act on the starch granule surface to release oligosaccharides when other starch-degrading enzymes were not present [26, 27]. AMY3 was also found participated in the plant response to stress, particularly osmotic stress by mediating starch degradation and controlling the light-induced stomatal opening in guard cells [13].

BAM proteins from cassava, rubber, castor, and jatropha in the Euphorbiaceae have tight evolutionary connections (Fig. 3b). MeBAM2, MeBAM7, and AtBAM1 were found in Group 7, while MeBAM4 and AtBAM2 were classified into Group 2. MeBAM9 and AtBAM3 were classified into Group 6. *AtBAM1* was found to be highly expressed in guard cells of leaves and the activity of the β-amylase encoded by *AtBAM1* was only active when high temperature, drought, and osmotic stress occurred [10]. Expression profiles of *MeBAM2* of cassava leaves were also found up-regulated under drought stress (Figs. 6b and 7a), which suggested that it can be one of the candidates genes response to drought. During the night, AtBAM3 was primarily in charge of starch breakdown within mesophyll cells [28]. Due to glutamic acid was changed to arginine at the catalytic active site Glu-380, the β-amylase encoded by *AtBAM4* lacked catalytic activity [29]. MeBAM8 shared the same catalytic active site as AtBAM4. Thus, the β-amylase encoded by MeBAM8 may also lack catalytic activity (Fig. S2b). Both MeBAM5 and MeBAM10 had a BZR1/BES1 domain at their N-termini as AtBAM7 and AtBAM8, which had been proved that played essential roles in bud growth and development by acting as transcription factors [30, 31]. However, the GO annotations did not adequately define transcription factor functions of MeBAM5 and MeBAM10. In addition, MeBAM1 was located in Group 5 with no Arabidopsis BAM (Fig. 3b) and Matthias Thalmann [32] suggested that this branch was formed before the seed plants radiation.

Under stress conditions, transcription factors combined with the cis-acting elements in the promoter region of stress-responsive genes and then promoted the transcription and expression of these genes, thus making a regulatory response [33]. In the current study, the *MeAMY* and *MeBAM* gene families included a range of stress response cis-acting elements and the largest number cis-acting elements were MYB-related elements, which is consistent with previous report [1]. MYB transcription factors are crucial for secondary metabolism, cell differentiation, stress response, and plant growth and development [34]. Under drought stress, most of *MeAMY* and *MeBAM* genes showed up-regulated expressions in cassava leaves (Fig. 6b), while less genes expressed up-regulated in the stems and roots (Fig. 7b and c). The majority of the *MeAMY* and *MeBAM* genes in cassava roots showed the highest expression level under moderate drought among three water treatments. The *MeAMY5* gene was highly expressed in stem and root under moderate drought. *MeAMY2* gene expression was stably up-regulated in roots, stems, and leaves (Fig. 7), while *MeAMY1* and *MeBAM3* gene expression was stably up-regulated in stems and leaves. Thus, these genes can be treated as critical genes in response to drought tolerance in cassava.

Metabolites of carbohydrates in plant leaves release soluble sugar, which can act as osmotic protecting agents, thus maintaining the water balance in cells enables plants to adapt to stress [13, 35–37]. During drought stress, the total enzyme activity of α-amylase and β-amylase increased in cassava leaves and stems, leading to higher soluble sugar and lower starch contents of cassava roots, stems, and leaves than those of the CK (Fig. 8), which is consistent with previous researches [13, 14, 28, 38, 39]. However, the activity of amylase in cassava roots under drought were decreased with the stress level (Fig. 8c) and similar phenomena were also found in tobacco (*Nicotiana tabacum*) and rape (*Brassica napus*) [35, 40]. These can be explained by previous findings. Starch hydrolysis process in roots might involve a number of synergistic amylases reactions [38, 41], leading to high maltose and soluble sugars contents than stem and leaf. And the high content of maltose inhibited the total enzyme activity of α-amylase and β-amylase [39]. In addition, it may also be a self-protection mechanism formed during the evolution of cassava. As the main energy storage organ of cassava, the starch preservation in the root during stress can provide energy for the rapid recovery of plant shoots after the stress is lifted.

## Conclusion

Ten *MeBAM* genes and six *MeAMY* genes were identified and characterized in the cassava genome. Both *MeAMY* and *MeBAM* gene families contain four genes with alternative splicing. According to domain analysis, MeBAM5 and MeBAM10 proteins may function as transcription factors. The promoter regions of *MeAMY* and *MeBAM* genes contain a large number of cis-acting elements related to abiotic stress. *MeAMY1*, *MeAMY2*, *MeAMY5*, and *MeBAM3* are critical genes in response to drought stress. These results provide fundamental knowledge for not only further exploring the starch metabolism functions of cassava under drought stress but also offering new perspectives for understanding the mechanism of how cassava survives and develops under drought.

## Methods

### Data resource for *AMY* and *BAM* gene families in cassava and other species

Cassava genome data were downloaded from Ensembl Plants Database (Manihot_esculenta_v6, http://plants.ensembl.org/Manihot_esculenta/Info/Index). The protein sequences of *AMY* and *BAM* genes of *Arabidopsis thaliana* and *Oryza sativa* were downloaded from NCBI (https://www.ncbi.nlm.nih.gov/) and Rice Genome Annotation Project (http://rice.uga.edu/index.shtml)(Table S2), respectively. The AMY and BAM sequences in the cassava database were characterized using the AMY and BAM proteins from Arabidopsis and rice as queries by TBtools-BLAST [42]. The local Hidden Markov Models of all the known AMY and BAM proteins of Arabidopsis and rice were constructed by matching their protein sequence dataset in HMMER (http://hmmer.org/). These models were employed to identify the candidate AMY and BAM proteins in cassava. And protein sequences with consistency ≥ 60% and E value ≤ 1e-5 were selected. The Pfam database (https://pfam.xfam.org/) and SMART (http://smart.embl-heidelberg.de/) database were then applied for searching the complete conserved domains of these selected sequences [43, 44]. The target *MeAMY* and *MeBAM* genes were identified by BLAST analyses on all candidate genes. The cassava protein sequences containing the Alpha-amylase domain (PF00128) and the Glyco_hydro_14 domain (PF01373) were treated as the *MeAMY* gene family sequences and the *MeBAM* gene family sequences, respectively.

### Characterization and subcellular localization prediction of MeAMY and MeBAM protein sequences

The relative molecular mass, instability index, grand average of hydropathicity (GRAVY), and isoelectric point (pI) of the identified MeAMY and MeBAM were predicted by the ExPASy-ProtParam platform (https://www.expasy.org/resources/protparam). Motifs of MeAMY and MeBAM were analyzed with MEME software (http://meme-suite.org/tools/meme) [45]. The optimum width of the motifs was set as 10 ~ 100, the number of motifs was set as 15, and the other parameters were used as default settings. The gene structure features of *MeAMY* and *MeBAM* were obtained based on the cassava genome GFF file. Similarity and composition analysis were done using software DNAMAN and BioEdit, respectively. Subcellular localizations of *MeAMY* and *MeBAM* were predicted by CELLO v. 2.5 (http://cello.life.nctu.edu.tw/) [46].

### Sequence alignment, phylogenetic analysis, and classification of MeAMY and MeBAM protein sequences

Multiple sequence alignment of MeAMY and MeBAM sequences was performed using DNAMAN and the alignment sequences were displayed using jalview (http://www.Jalview.org/). Un-rooted trees were constructed using the maximum likelihood method within MEGA-X software. The phylogenetic trees of AMY and BAM protein sequences of cassava and other species were built using the Neighbor-joining method, where the bootstrap was set to 1000 replicates.

### Chromosomal localization and collinearity analysis of *MeAMY* and *MeBAM*

The genome-wide protein database of cassava, Arabidopsis, and rubber trees was constructed by TBtools software. MCScanX (https://github.com/wyp1125/MCScanx) was used to analyze gene duplication and collinearity [47]. The collinearity map and chromosome mapping of homologous genes were plotted on chromosomes. The ratio of homologous genes Ka (nonsynonymous substitution rate) and Ks (synonymous substitution rate) was also calculated.

### Cis-regulatory elements analysis of *MeAMY* and *MeBAM* genes

The upstream regions comprising 2000 bp of translation start sites (initiation codon) of *MeAMY* and *MeBAM* sequences were downloaded as the promoter sequences from the Cassava Genome. The PlantCARE and New PLACE databases were used to predict the conserved cis-elements in these promoter sequences [48, 49].

### Gene ontology classification and expression analysis of *MeAMY* and *MeBAM* genes based on RNA-seq data

Gene ontology annotations of *MeAMY* and *MeBAM* genes were performed within the omicshare platform (https://www.omicshare.com/tools). The expression patterns of *MeAMY* and *MeBAM* genes were analyzed based on the raw RNA sequencing data, including different tissue types (study accession: SRP228273, SRP076160, and SRP354996, Table S4) [50], which was obtained from NCBI Sequencing Read Archive (https://www.ncbi.nlm.nih.gov/sra/). The RNA-seq data was first standardized in the form of TPM (transcripts per million reads) and then the log2(TPM + 1) conversion was performed before visualization. A heat map was created using ggplot2 in R 4.2.1.

### Plant materials and drought treatments

Cassava variety South China 205 (SC205) was used as plant material, which is the most popular cultivated in China because of its stable root yields and provided by Guangxi South Subtropical Agricultural Sciences Research Institute. The study was conducted at Guangxi University in 2021 and cassava was planted in a cuboid box (40 × 40 × 50 cm) with soil in October. Two cassava seedlings were planted in each box with normal watering and well management. The water treatment was set as three levels: 70 ~ 80% of soil water content as control (CK), 45 ~ 55% as moderate drought (MD), and 20 ~ 30% as severe drought (HD). And drought treatments were operated on 60 days after planting and lasted for one week. Each treatment contained six boxes and 12 shoots of cassava. A soil moisture recorder (LUGE-L99-TWS-1) was used to monitor the moisture content in the soil during the drought treatment period. After that, cassava stems (0 ~ 10 cm from the top of the stem), leaves, and roots samples were collected. Each sample was separated into two parts. One part was frozen in liquid nitrogen and stored at -80 °C for molecular and physiological analysis. The other part was oven-dried and ground for further determinations of the starch and sugar contents.

### Determination of contents of soluble sugar and starch and activities of amylase of cassava tissues

The protocol for analyzing the contents of soluble sugar and starch in root, stem, and leaf of cassava was described by Loewus [51]. Determination of activities of amylase in plant samples using the 3,5-dinitrosalicylic acid (DNS) colorimetric method and details were well described by Bernfeld [52].

### RNA extraction and qRT-PCR assays

Total RNA was extracted using the FastPure® Plant Total RNA Isolation Kit (Vazyme, China) and operated according to the manufacturer’s instructions. The first-strand cDNA was synthesized using a HiScript® II Q Select RT Super Mix for qPCR + gDNA wiper Kit (Vazyme). The housekeeping gene of cassava *actin* was used as an internal control to normalize the data. The primers were designed using the NCBI-Primer-BLAST tool (Table S5) and the range of the PCR products was set as 80 ~ 200 bp. qRT-PCR was carried out using ChamQ Universal SYBR qPCR Master Mix (Vazyme) in a qTOWER2.2 real-time PCR system (Analytik Jena, Germany) in accordance with the manufacturer’s protocol. The total volume of 10 µL qRT-PCR reaction was used, including 5 µL ChamQ Universal SYBR qPCR Master Mix, 0.4 µL of forward primer (10 µM), 0.4 µL of reverse primer (10 µM), 1 µL of cDNA, and 3.2 µL of ddH_2_O. The cycling conditions were 95 ℃ for 30 S, followed by 42 cycles at 95 ℃ for 10 S and 60 ℃ for 30 S. Melting curve analysis was then performed ranging from 60 to 95 ℃. The relative expression levels in CK and drought treatments were calculated with the formulas 2^−ΔCt^ and 2^−ΔΔCt^ [53], respectively. Three independent biological replicates were applied for all treatments. Statistical analysis was calculated using IBM SPSS 25.0.

## Electronic supplementary material

Below is the link to the electronic supplementary material.

**Additional file 1: Table S1.** Collinear gene pairs of *AMY* and *BAM* encoding genes among *Manihot esculenta*, *Arabidopsis thaliana*, and *Hevea brasiliensis.*

**Additional file 2: Table S2.** Accession numbers for AMY and BAM protein sequences in different plants.

**Additional file 3: Table S3.** GO annotation results of *MeAMY* and *MeBAM* genes.

**Additional file 4: Table S4.** RNA-seq data information of cassava.

**Additional file 5: Table S5.** Primers for qRT-PCR.

**Additional file 6: Fig. S1.** Similarities of *MeAMY* (a) and *MeBAM* (b) gene family sequences.

**Additional file 7: Fig. S2.** Alignment analysis of MeAMY and MeBAM protein sequences.

**Additional file 8: Fig. S3.** Chromosomal distribution of *MeAMY* and *MeBAM* genes.

**Additional file 9: Fig. S4.** Predicted cis-elements in the promoter regions of *MeAMY* (a) and *MeBAM* (b) genes.

**Additional file 10: Fig. S5.** Expression patterns of *MeAMY* and *MeBAM* genes in different parts of cassava under well watering condition.

## Data Availability

All data used in this study are publicly available and included in supplementary information files. *Manihot esculenta* Crantz genome sequences were obtained from the Ensembl Plants Database (Manihot_esculenta_v6, http://plants.ensembl.org/Manihot_esculenta/Info/Index), while transcriptomic sequencing data were downloaded from NCBI BioProject PRJNA578024, PRJNA324539, PRJNA796531(https://www.ncbi.nlm.nih.gov/bioproject/). Genomic data for *Hevea brasiliensis* (ASM165405v1) and *Arabidopsis thaliana* (TAIR10.1) were obtained from NCBI (https://www.ncbi.nlm.nih.gov/genome/).

## Abbreviations

AMY: α-amylases
BAM: β-amylases
Glyco_hydro_14: Glycoside hydrolase family 14
GFF: Generic Feature Forma
ABA: Abscisic acid
ROS: reactive oxygen species
ADS: Alternate donor site
AP: Alternate promoter
AT: Alternate terminator
GRAVY: Grand averages of hydropathicity
pI: Isoelectric points
qRT-PCR: Quantitative real-time polymerase chain reaction
Ka: nonsynonymous substitution rate
Ks: synonymous substitution rate
