# Supplementary material for: Genome-wide characterization and expression analysis of α-amylase and β-amylase genes underlying drought tolerance in cassava
Source: BMC Genomics. 2023 Apr 6;24:190. doi: 10.1186/s12864-023-09282-9 (PMC10080747; doi:10.1186/s12864-023-09282-9)
Supplement: Supplementary file 1 — Additional file 1: Table S1. Collinear gene pairs of AMY and BAM encoding genes among Manihot esculenta, Arabidopsis thaliana, and Hevea brasiliensis. [file 12864_2023_9282_MOESM1_ESM.pdf]

Table S1 Collinear gene pairs of *AMY* and *BAM* encoding genes among *Manihot esculenta*, *Arabidopsis thaliana*, and *Hevea brasiliensis*

Gene	Species	Protein accession	Protein accession
	Hevea brasiliensis	KAF2299582.1	XP_021603448.1(MeAMY1.1)
		KAF2302413.1	XP_021603448.1(MeAMY1.1)
	Arabidopsis thaliana	NP_564977.1(AtAMY3)	XP_021603448.1(MeAMY1.1)
		KAF2299598.1	XP_021611463.1(MeAMY2.1)
	Hevea brasiliensis	KAF2299582.1	XP_021611463.1(MeAMY2.1)
		KAF2302413.1	XP_021611463.1(MeAMY2.1)
	Arabidopsis thaliana	NP_564977.1(AtAMY3)	XP_021611463.1(MeAMY2.1)
AMY	*Manihot esculenta*	XP_021603448.1(MeAMY1.1)	XP_021611463.1(MeAMY2.1)
	Hevea brasiliensis	KAF2324746.1	XP_021612774.1(MeAMY3.1)
		KAF2310166.1	XP_021612774.1(MeAMY3.1)
	Arabidopsis thaliana	NP_001323338.1(AtAMY2)	XP_021612774.1(MeAMY3.1)
		KAF2283954.1	XP_021631425.1(MeAMY5)
	Hevea brasiliensis	KAF2292976.1	XP_021631425.1(MeAMY5)
		KAF2309820.1	XP_021601526.1(MeAMY6.1)
		KAF2313488.1	XP_021605553.1(MeBAM1)
	Hevea brasiliensis	KAF2285574.1	XP_021608087.1(MeBAM2)
		KAF2314082.1	XP_021608087.1(MeBAM2)
		KAF2314091.1	XP_021608087.1(MeBAM2)
	Arabidopsis thaliana	NP_189034.1(AtBAM1)	XP_021608087.1(MeBAM2)
	Hevea brasiliensis	KAF2311005.1	XP_021608216.1(MeBAM3)
	Arabidopsis thaliana	NP_197368.1(AtBAM3)	XP_021608216.1(MeBAM3)
	Hevea brasiliensis	KAF2282586.1	XP_021611624.1(MeBAM4.1)
	Arabidopsis thaliana	NP_182112.2(AtBAM7)	XP_021611624.1(MeBAM4.1)
BAM		NP_191958.3(AtBAM2)	XP_021611624.1(MeBAM4.1)
		KAF2315311.1	XP_021613139.1(MeBAM5.1)
	Hevea brasiliensis	KAF2285574.1	XP_021594996.1(MeBAM7)
		KAF2314082.1	XP_021594996.1(MeBAM7)
	Manihot esculenta	XP_021608087.1(MeBAM2)	XP_021594996.1(MeBAM7)
		XP_021611624.1(MeBAM4)	XP_021613139.1(MeBAM5)
	Hevea brasiliensis	KAF2288954.1	XP_021595073.1(MeBAM8)
		KAF2305562.1	XP_021595073.1(MeBAM8)
	Arabidopsis thaliana	NP_568829.2(AtBAM4)	XP_021595073.1(MeBAM8)
	Hevea brasiliensis	KAF2308349.1	XP_021594434.1(MeBAM9)
		KAF2316086.1	XP_021594905.1(MeBAM10.1)
