# Supplementary material for: Genome-wide characterization and expression analysis of α-amylase and β-amylase genes underlying drought tolerance in cassava
Source: BMC Genomics. 2023 Apr 6;24:190. doi: 10.1186/s12864-023-09282-9 (PMC10080747; doi:10.1186/s12864-023-09282-9)
Supplement: Supplementary file 2 — Additional file 2: Table S2. Accession numbers for AMY and BAM protein sequences in different plants. [file 12864_2023_9282_MOESM2_ESM.pdf]

Table S2 Accession numbers for AMY and BAM protein sequences in different plants

Gene	Species	Accession	Gene	Species	Accession
		AAA32929.1			CAA67128.1
	Triticum aestivum	AAA98790.1		*Triticum aestivum*	SPT17452.1
		AAA32932.1			SPT17453.1
		AAA32933.1			SPT16472.1
	Beta vulgaris	KMS99661.1		*Camellia sinensis*	AKQ62958.1
		KMS99660.1		*Ziziphus jujuba*	XP_024928937.1
	Daucus carota	KZM86731.1		*Vitis riparia*	XP_034710933.1
		KZM93500.1		*Theobroma cacao*	EOY12027.1
		KZM85190.1			XP_006339564.1
		KZM88353.1			XP_006340896.1
	Hevea brasiliensis	KAF2321179.1			XP_006342739.1
		AID51355.1		*Solanum tuberosum*	XP_006362484.1
		XP_021655335.1			XP_006343811.1
	Ipomoea batatas	ALS87700.1			XP_006360595.1
	Malus domestica	XP_008373148.2			AAK84008.1
AMY	*Pistacia vera*	XP_031267192.1	*BAM*	*Salix suchowensis*	KAG5228777.1
	Quercus suber	XP_023913396.1			EEF50527.1
		EEF52808.1			EEF45479.1
	Ricinus communis	EEF36314.1			EEF42523.1
		EEF42189.1		*Ricinus communis*	EEF39599.1
		EEF52405.1			EEF46659.1
		AAA91884.1			EEF50526.1
		XP_006342097.1			EEF44329.1
		ACZ26470.1			EEF45055.1
	Solanum tuberosum	ACV30014.1		*Quercus suber*	XP_023873982.1
		XP_006354888.1		*Populus trichocarpa*	XP_006385389.1
		AAA91883.1		*Populus alba*	XP_034888216.1
		XP_015168383.1			XP_034914681.1
	Tripterygium wilfordii	XP_038704229.1		*Jatropha curcas*	KDP28061.1
	Vitis riparia	XP_034681991.1			KDP40025.1
	Arabidopsis	AEE34983.1			KDP33360.1

Gene	Species	Accession	Gene	Species	Accession
	thaliana	AEE84990.1			KDP28630.1
		AEE35800.1			KDP25878.1
		BAD17125.1			KDP45986.1
		BAD09334.1			KDP25257.1
		BAD09335.1			KDP39273.1
AMY	*Oryza sativa*	BAD38369.1		*Ipomoea batatas*	BAA02286.1
		BAD38368.1			XP_021635656.1
		BAD38366.1			XP_021678144.1
		BAD54103.1			XP_021653095.1
		BAD52958.1			XP_021669733.1
	Gossypium hirsutum	XP_040963198.1			XP_021689660.1
	Herrania umbratica	XP_021291887.1		*Hevea brasiliensis*	XP_021674698.1
		XP_021653095.1	*BAM*		XP_021636026.1
	Citrus clementina	XP_006420416.1			XP_021291887.1
	Carica papaya	XP_021904320.1			XP_021281295.1
BAM		LOC_Os01g13550.1		*Durio zibethinus*	XP_022716740.1
		LOC_Os02g03690.1			Q9LIR6.1
		LOC_Os03g04770.1			O23553.3
		LOC_Os03g22790.1			P25853.1
	Oryza sativa	LOC_Os07g35880.1			O65258.2
		LOC_Os07g35940.1		*Arabidopsis thaliana*	O80831.2
		LOC_Os07g47120.1			Q9FH80.1
		LOC_Os09g39570.1			Q9FM68.1
		LOC_Os10g32810.1			Q8L762.1
		LOC_Os10g41550.1			Q8VYW2.1
