# Supplementary material for: Genome-wide characterization and expression analysis of α-amylase and β-amylase genes underlying drought tolerance in cassava
Source: BMC Genomics. 2023 Apr 6;24:190. doi: 10.1186/s12864-023-09282-9 (PMC10080747; doi:10.1186/s12864-023-09282-9)
Supplement: Supplementary file 3 — Additional file 3: Table S3. GO annotation results of MeAMY and MeBAM genes. [file 12864_2023_9282_MOESM3_ESM.pdf]

Table S3 GO annotation results of *MeAMY* and *MeBAM* genes.

ID	Description	Number	Qvalue	Ratio
GO:0016160	amylase activity	16	8.61E-52	0.842
GO:0016161	beta-amylase activity	10	4.29E-31	0.909
GO:0000272	polysaccharide catabolic process	15	8.31E-26	0.065
GO:0004553	hydrolase activity, hydrolyzing O-glycosyl compounds	16	1.77E-23	0.024
GO:0016798	hydrolase activity, acting on glycosyl bonds	16	4.79E-23	0.022
GO:0016052	carbohydrate catabolic process	15	8.91E-21	0.029
GO:0005976	polysaccharide metabolic process	15	7.81E-20	0.025
GO:0004556	alpha-amylase activity	6	1.37E-17	0.75
GO:0009057	macromolecule catabolic process	15	1.14E-16	0.015
GO:0008788	alpha,alpha-phosphotrehalase activity	6	2.04E-15	0.4
GO:0047471	maltose alpha-D-glucosyltransferase activity	6	2.8E-15	0.375
GO:0015927	trehalase activity	6	3.78E-15	0.353
GO:0005975	carbohydrate metabolic process	16	1.15E-14	0.008
GO:0047470	(1,4)-alpha-D-glucan 1-alpha-D-glucosylmutase activity	5	3.91E-13	0.417
GO:1901575	organic substance catabolic process	15	4.9E-12	0.007
GO:0004133	glycogen debranching enzyme activity	5	6.86E-12	0.25
GO:0009056	catabolic process	15	7.54E-12	0.007
GO:0005992	trehalose biosynthetic process	6	1.54E-11	0.14
GO:0005991	trehalose metabolic process	6	2.1E-11	0.13
GO:0005980	glycogen catabolic process	5	4.87E-11	0.25
GO:0046351	disaccharide biosynthetic process	6	5.92E-11	0.107
GO:0044247	cellular polysaccharide catabolic process	5	6.89E-11	0.227
GO:0009251	glucan catabolic process	5	8.12E-11	0.217
GO:0016787	hydrolase activity	16	3.18E-10	0.003
GO:0051060	pullulanase activity	4	6.16E-10	0.286
GO:0005984	disaccharide metabolic process	6	3.33E-09	0.054
GO:0016866	intramolecular transferase activity	6	2.21E-08	0.028
GO:0005977	glycogen metabolic process	5	8.86E-08	0.056
GO:0044275	cellular carbohydrate catabolic process	5	0.000000181	0.048
GO:0009312	oligosaccharide biosynthetic process	6	0.000000187	0.027
GO:0005509	calcium ion binding	6	0.000000202	0.019
GO:0003844	1,4-alpha-glucan branching enzyme activity	3	0.000000344	0.2
GO:0006112	energy reserve metabolic process	5	0.000000419	0.039
GO:0009311	oligosaccharide metabolic process	6	0.000000537	0.022
GO:0034637	cellular carbohydrate biosynthetic process	6	0.00000215	0.017
GO:0046527	glucosyltransferase activity	4	0.00000228	0.037
GO:0006073	cellular glucan metabolic process	5	0.00000345	0.025
GO:0044042	glucan metabolic process	5	0.00000345	0.025
GO:0009250	glucan biosynthetic process	4	0.0000135	0.034
GO:0016051	carbohydrate biosynthetic process	6	0.0000142	0.012
GO:0044724	single-organism carbohydrate catabolic process	5	0.0000218	0.017

ID	Description	Number	Qvalue	Ratio
GO:0044262	cellular carbohydrate metabolic process	6	0.0000264	0.011
GO:0016853	isomerase activity	6	0.0000469	0.007
GO:0044264	cellular polysaccharide metabolic process	5	0.0000797	0.012
GO:0005978	glycogen biosynthetic process	3	0.00012411	0.041
GO:0015980	energy derivation by oxidation of organic compounds	5	0.000138674	0.011
GO:0016758	transferase activity, transferring hexosyl groups	4	0.000276499	0.011
GO:0003824	catalytic activity	16	0.000508286	0.001
GO:0033692	cellular polysaccharide biosynthetic process	4	0.000514421	0.013
GO:0000271	polysaccharide biosynthetic process	4	0.000547762	0.012
GO:0006091	generation of precursor metabolites and energy	5	0.000695721	0.008
GO:0044723	single-organism carbohydrate metabolic process	6	0.000975016	0.005
GO:0043170	macromolecule metabolic process	15	0.001871748	0.002
GO:0044238	primary metabolic process	16	0.004758722	0.001
GO:0016757	transferase activity, transferring glycosyl groups	4	0.004835155	0.005
GO:0044712	single-organism catabolic process	5	0.006678222	0.005
GO:0071704	organic substance metabolic process	16	0.009460981	0.001
GO:0046872	metal ion binding	6	0.022468859	0.002
GO:0043169	cation binding	6	0.023362477	0.002
GO:0044248	cellular catabolic process	5	0.03318996	0.003
GO:0008152	metabolic process	16	0.05099982	0.001
GO:0045182	translation regulator activity	1	0.094121009	0.01
GO:0044711	single-organism biosynthetic process	6	0.100761142	0.002
GO:0055114	oxidation-reduction process	5	0.143913855	0.002
GO:0006417	regulation of translation	1	0.289818083	0.006
GO:0034248	regulation of cellular amide metabolic process	1	0.289818083	0.006
GO:0010608	posttranscriptional regulation of gene expression	1	0.295608805	0.006
GO:0032268	regulation of cellular protein metabolic process	1	0.384190072	0.004
GO:0051246	regulation of protein metabolic process	1	0.384190072	0.004
GO:0043167	ion binding	6	0.540271435	0.001
GO:0016740	transferase activity	4	0.730892681	0.001
GO:0034645	cellular macromolecule biosynthetic process	4	0.964648894	0.001
GO:0009059	macromolecule biosynthetic process	4	0.964648894	0.001
GO:0044249	cellular biosynthetic process	6	1	0.001
GO:1901576	organic substance biosynthetic process	6	1	0.001
GO:0009058	biosynthetic process	6	1	0.001
GO:0006412	translation	1	1	0.001
GO:0044710	single-organism metabolic process	6	1	0.001
GO:0043043	peptide biosynthetic process	1	1	0.001
GO:0006518	peptide metabolic process	1	1	0.001
GO:0043604	amide biosynthetic process	1	1	0.001
GO:0043603	cellular amide metabolic process	1	1	0.001
GO:0010468	regulation of gene expression	1	1	0.001
GO:0044763	single-organism cellular process	6	1	0.001

ID	Description	Number	Qvalue	Ratio
GO:2000112	regulation of cellular macromolecule biosynthetic process	1	1	0.001
GO:0010556	regulation of macromolecule biosynthetic process	1	1	0.001
GO:0031326	regulation of cellular biosynthetic process	1	1	0.001
GO:0009889	regulation of biosynthetic process	1	1	0.001
GO:0051171	regulation of nitrogen compound metabolic process	1	1	0.001
GO:0031323	regulation of cellular metabolic process	1	1	0
GO:0080090	regulation of primary metabolic process	1	1	0
GO:0060255	regulation of macromolecule metabolic process	1	1	0
GO:0019222	regulation of metabolic process	1	1	0
GO:0050794	regulation of cellular process	1	1	0
GO:0050789	regulation of biological process	1	1	0
GO:0044260	cellular macromolecule metabolic process	5	1	0.001
GO:0065007	biological regulation	1	1	0
GO:1901566	organonitrogen compound biosynthetic process	1	1	0
GO:0044699	single-organism process	6	1	0.001
GO:1901564	organonitrogen compound metabolic process	1	1	0
GO:0010467	gene expression	1	1	0
GO:0044267	cellular protein metabolic process	1	1	0
GO:0005488	binding	6	1	0
GO:0044271	cellular nitrogen compound biosynthetic process	1	1	0
GO:0044237	cellular metabolic process	6	1	0.001
GO:0019538	protein metabolic process	1	1	0
GO:0034641	cellular nitrogen compound metabolic process	1	1	0
GO:0006807	nitrogen compound metabolic process	1	1	0
GO:0009987	cellular process	6	1	0
GO:0003674	molecular_function	16	1	0.001
GO:0008150	biological_process	16	1	0.001
