# Supplementary material for: Genome-wide characterization and expression analysis of α-amylase and β-amylase genes underlying drought tolerance in cassava
Source: BMC Genomics. 2023 Apr 6;24:190. doi: 10.1186/s12864-023-09282-9 (PMC10080747; doi:10.1186/s12864-023-09282-9)
Supplement: Supplementary file 4 — Additional file 4: Table S4. RNA-seq data information of cassava. [file 12864_2023_9282_MOESM4_ESM.pdf]

Table S4 RNA-seq data information of cassava

Accession	Sample information	Replicate	Rename
SRR10480846	100 days old stem	biological replicate 2	Stem(100d)
SRR10480847		biological replicate 1	
SRR10480905		biological replicate 3	
SRR10480848	100 days old leaf	biological replicate 3	Leaf(100d)
SRR10480851		biological replicate 2	
SRR10480862		biological replicate 1	
SRR10480873	100 days old fibrous root	biological replicate 3	Fibrous root(100d)
SRR10480884		biological replicate 2	
SRR10480895		biological replicate 1	
SRR10480882	340 days old storage root	biological replicate 3	Tuberous root(340d)
SRR10480883		biological replicate 2	
SRR10480885		biological replicate 1	
SRR10480886	300 days old storage root	biological replicate 3	Tuberous root(300d)
SRR10480887		biological replicate 2	
SRR10480888		biological replicate 1	
SRR10480889	260 days old storage root	biological replicate 3	Tuberous root(260d)
SRR10480890		biological replicate 2	
SRR10480891		biological replicate 1	
SRR10480892	220 days old storage root	biological replicate 3	Tuberous root(220d)
SRR10480893		biological replicate 2	
SRR10480894		biological replicate 1	
SRR10480896	180 days old storage root	biological replicate 3	Tuberous root(180d)
SRR10480897		biological replicate 2	
SRR10480898		biological replicate 1	
SRR10480899	140 days old storage root	biological replicate 3	Tuberous root(140d)
SRR10480900		biological replicate 2	
SRR10480901		biological replicate 1	
SRR10480902	100 days old storage root	biological replicate 3	Tuberous root(100d)
SRR10480903		biological replicate 2	
SRR10480904		biological replicate 1	
SRR3629821	Petiole of cassava	biological replicate 3	Petiole
SRR3629838		biological replicate 2	
SRR3629856		biological replicate 1	
SRR3629822	The bud of cassava	biological replicate 3	Bud
SRR3629840		biological replicate 2	
SRR3629858		biological replicate 1	
SRR17615134	Cassava leaves under normal conditions	biological replicate 3	Control
SRR17615135		biological replicate 2	
SRR17615136		biological replicate 1	
SRR17615124	Cassava mature leaves treated with drought for 5 days	biological replicate 3	Drought stress(5d)
SRR17615125		biological replicate 2	

Accession	Sample information	Replicate	Rename
SRR17615126	Cassava mature leaves treated with drought for 10 days	biological replicate 1	Drought stress(10d)
SRR17615121		biological replicate 3	
SRR17615122		biological replicate 2	
SRR17615123		biological replicate 1	
