# Supplementary material for: Genome-wide characterization and expression analysis of α-amylase and β-amylase genes underlying drought tolerance in cassava
Source: BMC Genomics. 2023 Apr 6;24:190. doi: 10.1186/s12864-023-09282-9 (PMC10080747; doi:10.1186/s12864-023-09282-9)
Supplement: Supplementary file 5 — Additional file 5: Table S5. Primers for qRT-PCR. [file 12864_2023_9282_MOESM5_ESM.pdf]

Table S5 Primers for qRT-PCR

Gene name	Primer ID	Primer sequences(5'-3')
MeAMY1	MeAMY1.1-F	AGCGTTGGCAGCTTAGTGT
	MeAMY1.1-R	ATAGGAACAGAACCTGGGGGT
MeAMY2	MeAMY2.1-F	TGTTGGCAGCTTAGCGTAGG
	MeAMY2.1-R	ACAGGAATAGAACCAGGGGGT
MeAMY3	MeAMY3.1-F	ATGGAGGCAACTGCGTGATT
	MeAMY3.1-R	AGCGCCAAAGCTAAGAAGGT
MeAMY4	MeAMY4.1-F	TTGAAGGTGAGCTATGGCGG
	MeAMY4.1-R	GACACTACTCCCTGGCAACA
MeAMY5	MeAMY5.1-F	TTTTGGCCGCCGATTCTGAT
	MeAMY5.1-R	TTCCAGAGGCAACAACCTGA
MeAMY6	MeAMY6.1-F	ATGCCGTGTGGGGTTTCAAT
	MeAMY6.1-R	AGGTGAAACTGACTGAGAGGG
MeBAM1	MeBAM1-F	TCAGATGATGCAATTTGGGGC
	MeBAM1-R	ATAAGATTTGCCTTGCGGGAG
MeBAM2	MeBAM2-F	AAGTGAATCTACGGCCACGG
	MeBAM2-R	TACTGGAAACGGACAAGGGC
MeBAM3	MeBAM3-F	TGGGGAATAGCCGAGAAGGA
	MeBAM3-R	CGTGGAGTTTGAGACCAGCA
MeBAM4	MeBAM4-F	CCTGTCCTTCGTAGCTTCCAT
	MeBAM4-R	ACGAGACCACCGCTAAAACA
MeBAM5	MeBAM5-F	TCTCTCCTGGTTATCGGCCT
	MeBAM5-R	CCATCCCCCATCATTGCTGA
MeBAM6	MeBAM6-F	ATGAGCTTGGGAAGCAGCTC
	MeBAM6-R	TACGATCCCCCACCAGACAT
MeBAM7	MeBAM7-F	ACAGGACCCATCACCACAAC
	MeBAM7-R	TCGGCTGAGATTTCTGCTCC
MeBAM8	MeBAM8-F	AATGGAGGAGTTGCTCGCAG
	MeBAM8-R	CGTTTCGGAGATTGGTTCGC
MeBAM9	MeBAM9-F	TCATGCCGCCTTAGAGCAAA
	MeBAM9-R	ACACAGGCACCTTAGCAACA
MeBAM10	MeBAM10-F	TCAACAGCCATCACCTTTGGT
	MeBAM10-R	CTGAACTCACTGCTGGGGT
actin	actin-F	TGATGAGTCTGGTCCATCCA
	actin-R	CCTCCTACGACCCAATCTCA
