# Supplementary figures and images for: Genome-wide characterization and expression analysis of α-amylase and β-amylase genes underlying drought tolerance in cassava
Source: BMC Genomics. 2023 Apr 6;24:190. doi: 10.1186/s12864-023-09282-9 (PMC10080747; doi:10.1186/s12864-023-09282-9)

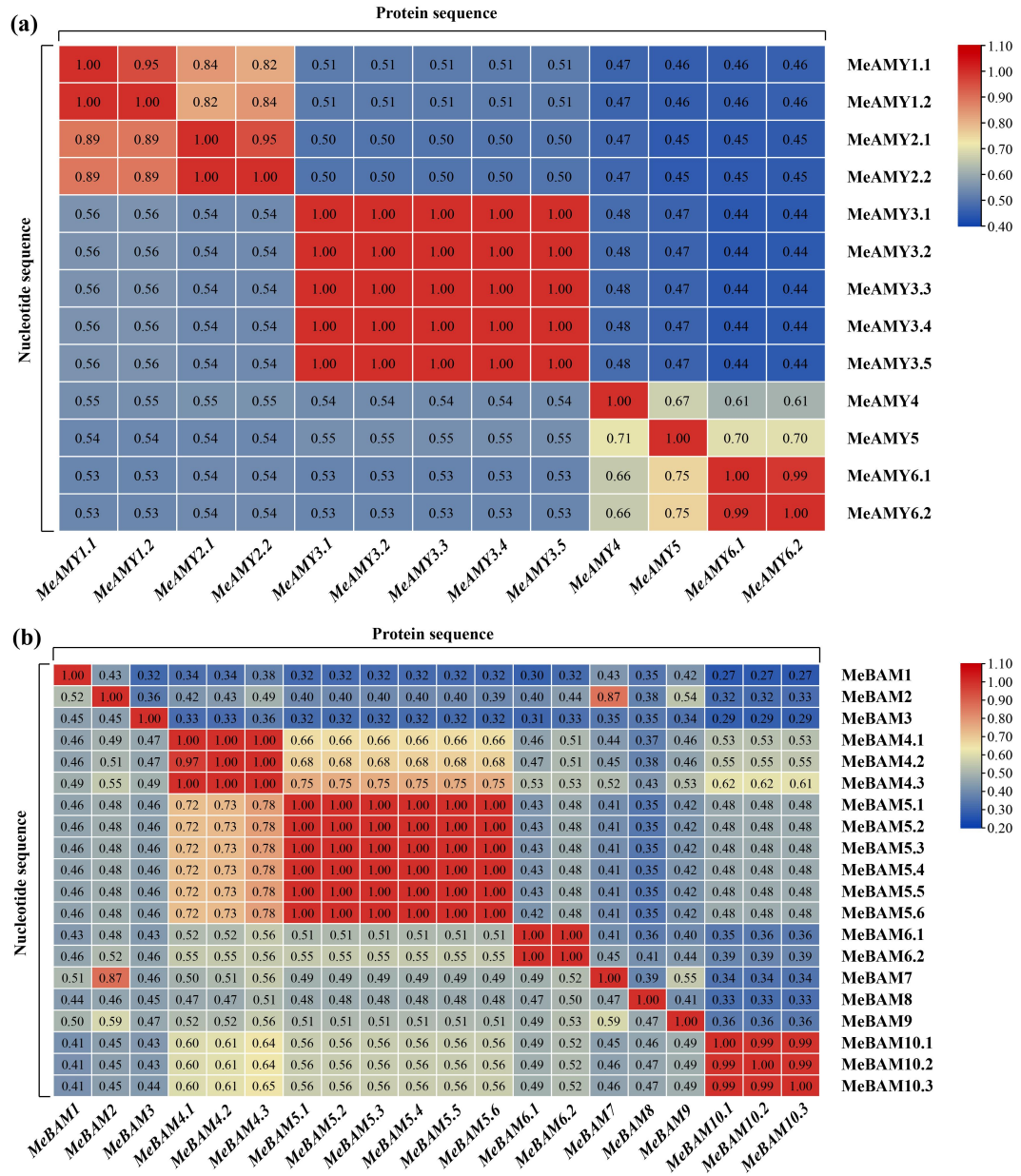

**Fig. S1** Similarities of *MeAMY* (a) and *MeBAM* (b) gene family sequences.

Supplement: Supplementary file 6 — Additional file 6: Fig. S1. Similarities of MeAMY (a) and MeBAM (b) gene family sequences. [file 12864_2023_9282_MOESM6_ESM.pdf]

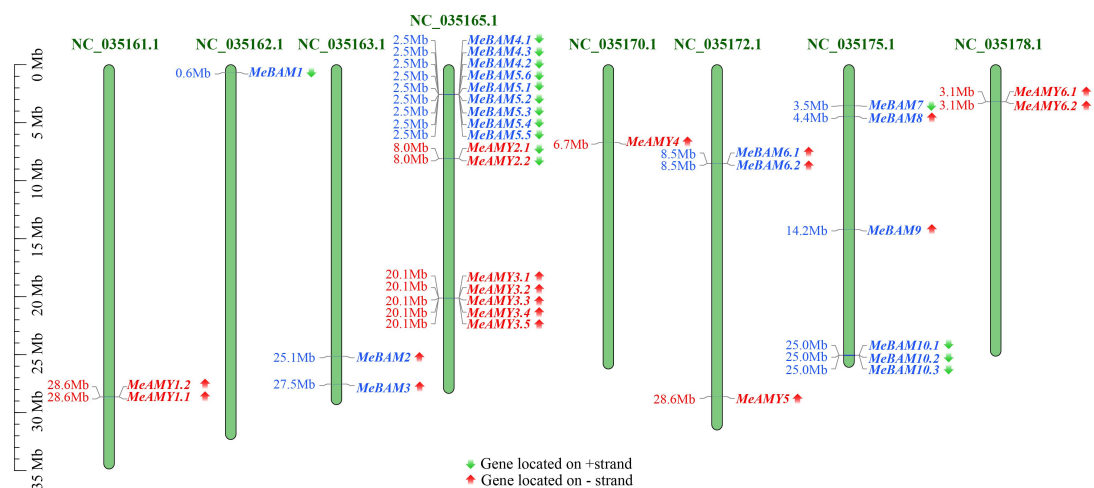

**Fig. S3** Chromosomal distribution of *MeAMY* and *MeBAM* genes.

Supplement: Supplementary file 8 — Additional file 8: Fig. S3. Chromosomal distribution of MeAMY and MeBAM genes. [file 12864_2023_9282_MOESM8_ESM.pdf]
