# Supplementary material for: Genome-wide characterization and expression analysis of α-amylase and β-amylase genes underlying drought tolerance in cassava
Source: BMC Genomics. 2023 Apr 6;24:190. doi: 10.1186/s12864-023-09282-9 (PMC10080747; doi:10.1186/s12864-023-09282-9)
Supplement: Supplementary file 9 — Additional file 9: Fig. S4. Predicted cis-elements in the promoter regions of MeAMY (a) and MeBAM (b) genes. [file 12864_2023_9282_MOESM9_ESM.pdf]

(a)

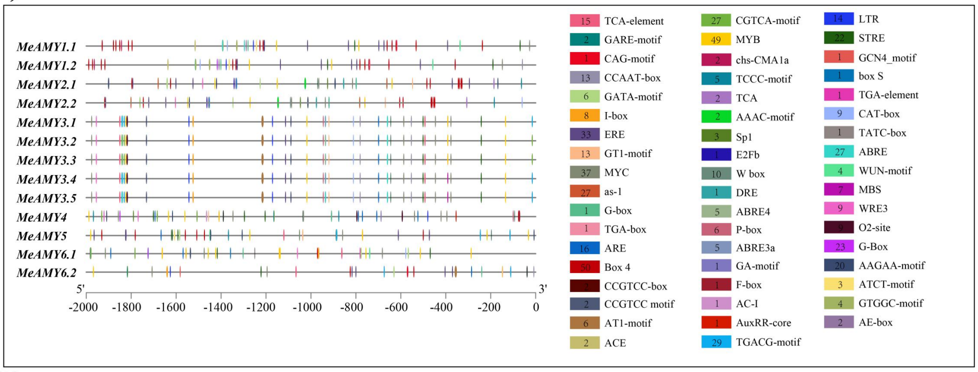

(b)

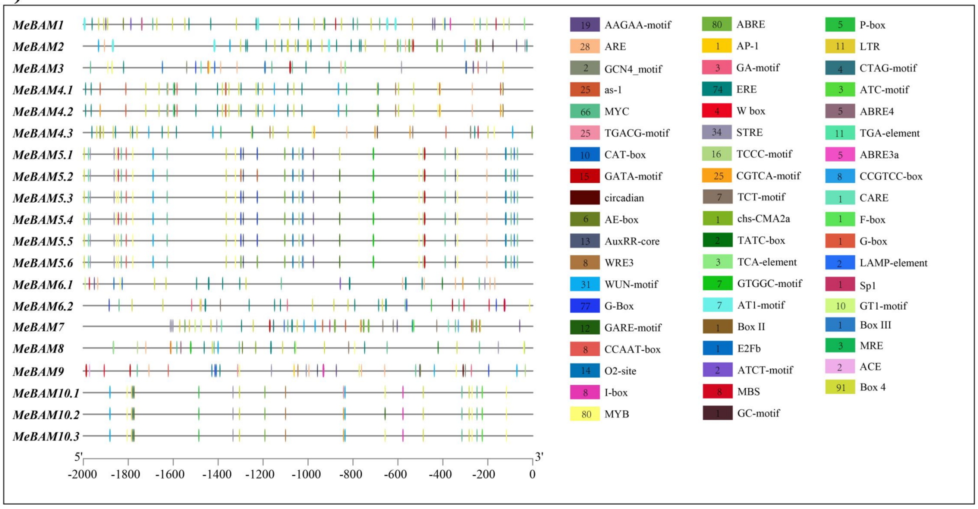

Fig. S4 Predicted cis-elements in the promoter regions of *MeAMY* **(a)**and *MeBAM* **(b)** genes. Numbers in color blocks represent the number of cis-acting elements.
