# Supplementary material for: Genome-wide characterization and expression analysis of α-amylase and β-amylase genes underlying drought tolerance in cassava
Source: BMC Genomics. 2023 Apr 6;24:190. doi: 10.1186/s12864-023-09282-9 (PMC10080747; doi:10.1186/s12864-023-09282-9)
Supplement: Supplementary file 10 — Additional file 10: Fig. S5. Expression patterns of MeAMY and MeBAM genes in different parts of cassava under well watering condition. [file 12864_2023_9282_MOESM10_ESM.pdf]

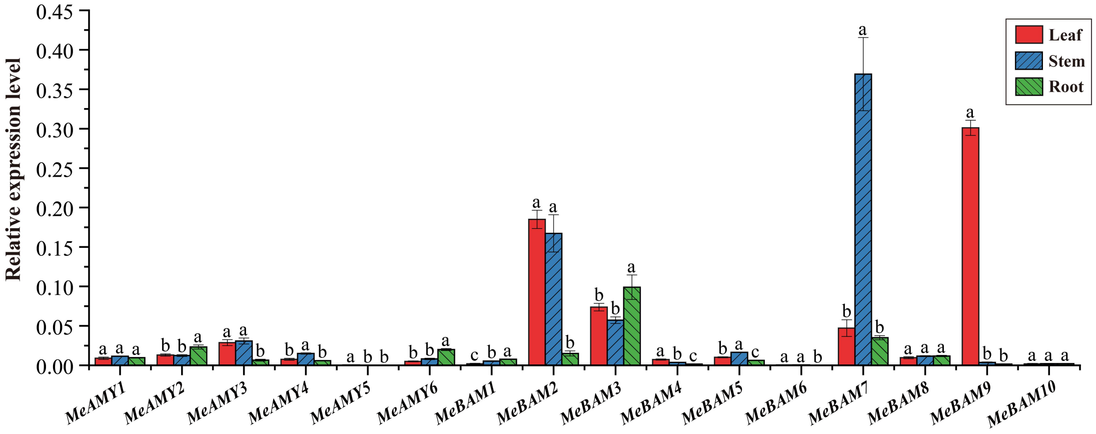

Fig. S5 Expression patterns of *MeAMY* and *MeBAM* genes in different parts of cassava under well watering condition. Expression levels were determined using qRT-PCR and calculated using the $2^{-\Delta Ct}$ method under the control of the *actin* housekeeping gene. Data are expressed as the mean ± SE of three independent replicates. Different letters indicate significant differences among different tissues of cassava ($P < 0.05$).
