# Supplementary material for: Genome-wide characterization and expression analysis of α-amylase and β-amylase genes underlying drought tolerance in cassava
Source: BMC Genomics. 2023 Apr 6;24:190. doi: 10.1186/s12864-023-09282-9 (PMC10080747; doi:10.1186/s12864-023-09282-9)
Supplement: Supplementary file 7 — Additional file 7: Fig. S2. Alignment analysis of MeAMY and MeBAM protein sequences. [file 12864_2023_9282_MOESM7_ESM.pdf]

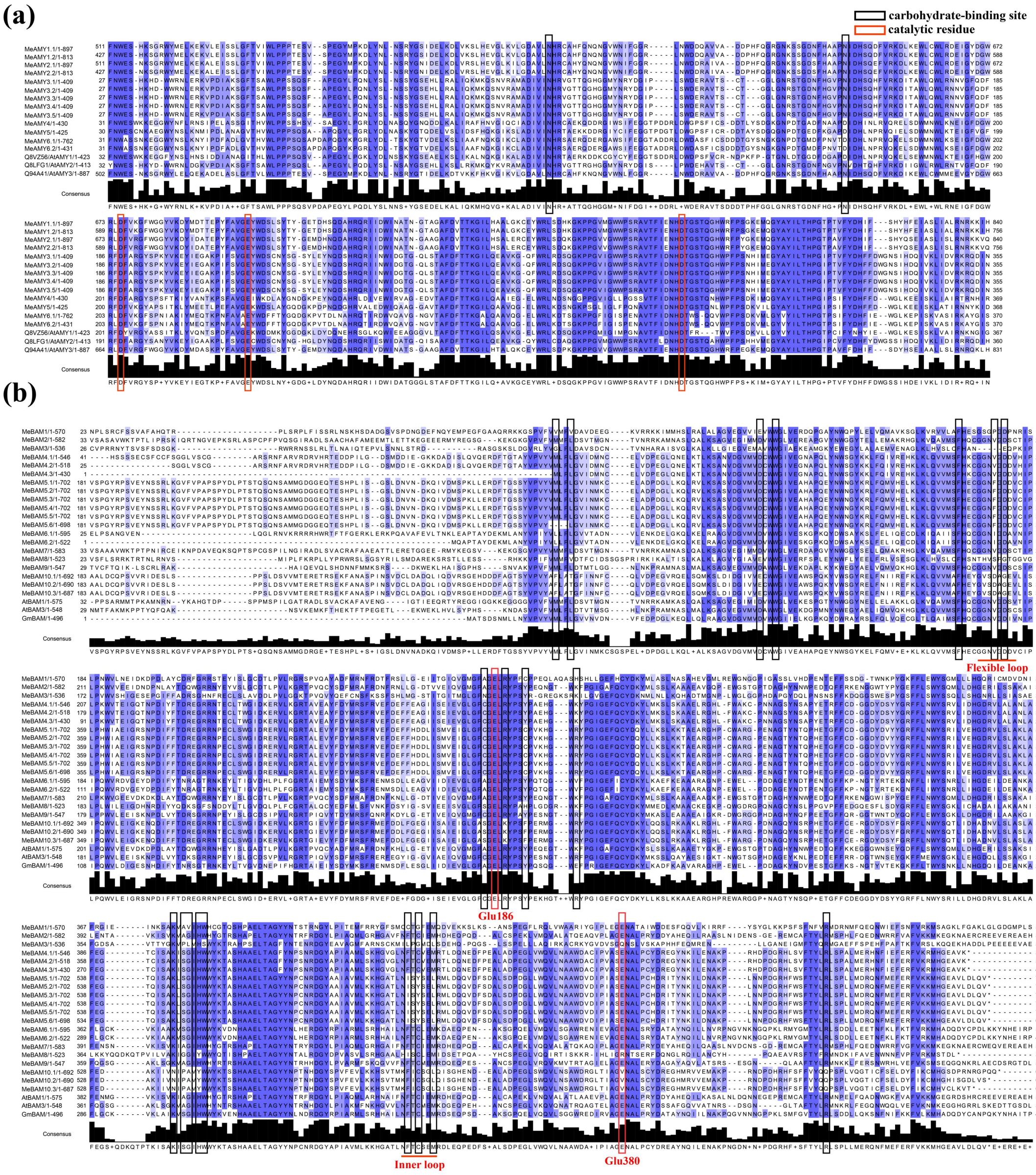

Fig. S2 Alignment analysis of MeAMY and MeBAM protein sequences. **a** Multiple sequence alignment of MeAMY family amino acid. **b** Multiple sequence alignment of MeBAM family amino acid. blue shading indicates highly conservative substitutions. Red box represents the two catalytic residues, black box represents carbohydrate binding site.
